# *Caenorhabditis elegans* RIG-I Homolog Mediates Antiviral RNA Interference Downstream of Dicer-Dependent Biogenesis of Viral Small Interfering RNAs

**DOI:** 10.1128/mBio.00264-17

**Published:** 2017-03-21

**Authors:** Stephanie R. Coffman, Jinfeng Lu, Xunyang Guo, Jing Zhong, Hongshan Jiang, Gina Broitman-Maduro, Wan-Xiang Li, Rui Lu, Morris Maduro, Shou-wei Ding

**Affiliations:** a Department of Plant Pathology and Microbiology, University of California, Riverside, California, USA; b Graduate Program in Genetics, Genomics and Bioinformatics, University of California, Riverside, California, USA; c Biology Department, University of California, Riverside, California, USA; d Department of Biological Sciences, Louisiana State University, Baton Rouge, Louisiana, USA; Mailman School of Public Health, Columbia University

**Keywords:** *Caenorhabditis elegans*, DRH-1, RNA interference, antiviral defense

## Abstract

Dicer enzymes process virus-specific double-stranded RNA (dsRNA) into small interfering RNAs (siRNAs) to initiate specific antiviral defense by related RNA interference (RNAi) pathways in plants, insects, nematodes, and mammals. Antiviral RNAi in *Caenorhabditis elegans* requires Dicer-related helicase 1 (DRH-1), not found in plants and insects but highly homologous to mammalian retinoic acid-inducible gene I (RIG-I)-like receptors (RLRs), intracellular viral RNA sensors that trigger innate immunity against RNA virus infection. However, it remains unclear if DRH-1 acts analogously to initiate antiviral RNAi in *C. elegans*. Here, we performed a forward genetic screen to characterize antiviral RNAi in *C. elegans*. Using a mapping-by-sequencing strategy, we uncovered four loss-of-function alleles of *drh-1*, three of which caused mutations in the helicase and C-terminal domains conserved in RLRs. Deep sequencing of small RNAs revealed an abundant population of Dicer-dependent virus-derived small interfering RNAs (vsiRNAs) in *drh-1* single and double mutant animals after infection with Orsay virus, a positive-strand RNA virus. These findings provide further genetic evidence for the antiviral function of DRH-1 and illustrate that DRH-1 is not essential for the sensing and Dicer-mediated processing of the viral dsRNA replicative intermediates. Interestingly, vsiRNAs produced by *drh-1* mutants were mapped overwhelmingly to the terminal regions of the viral genomic RNAs, in contrast to random distribution of vsiRNA hot spots when DRH-1 is functional. As RIG-I translocates on long dsRNA and DRH-1 exists in a complex with Dicer, we propose that DRH-1 facilitates the biogenesis of vsiRNAs in nematodes by catalyzing translocation of the Dicer complex on the viral long dsRNA precursors.

## IMPORTANCE

The helicase and C-terminal domains of mammalian RLRs sense intracellular viral RNAs to initiate the interferon-regulated innate immunity against RNA virus infection. Both of the domains from human RIG-I can substitute for the corresponding domains of DRH-1 to mediate antiviral RNAi in *C. elegans*, suggesting an analogous role for DRH-1 as an intracellular dsRNA sensor to initiate antiviral RNAi. Here, we developed a forward genetic screen for the identification of host factors required for antiviral RNAi in *C. elegans*. Characterization of four distinct *drh-1* mutants obtained from the screen revealed that DRH-1 did not function to initiate antiviral RNAi. We show that DRH-1 acted in a downstream step to enhance Dicer-dependent biogenesis of viral siRNAs in *C. elegans*. As mammals produce Dicer-dependent viral siRNAs to target RNA viruses, our findings suggest a possible role for mammalian RLRs and interferon signaling in the biogenesis of viral siRNAs.

## INTRODUCTION

Diverse eukaryotic hosts recognize and process virus-specific double-stranded RNA (dsRNA) into small interfering RNAs (siRNAs), which subsequently direct specific antiviral immunity by RNA interference (RNAi) (1, 2). The virus-derived siRNAs (vsiRNAs) are products of the Dicer family of dsRNA-specific endoribonucleases and therefore exhibit several unique biochemical properties. For example, vsiRNAs are 21 to 24 nucleotides (nt) in length, contain monophosphates at the 5′ ends, and form perfectly base-paired RNA duplexes with 2-nt 3′ overhangs. In addition to serving as a molecular marker for the induction of antiviral RNAi, vsiRNAs also function as the specificity determinants of the antiviral immunity after they are assembled with an Argonaute protein and cofactors into RNA-induced silencing complex (RISC) (1, 2).

Recent studies have established *Caenorhabditis elegans* as a small-animal model for antiviral RNAi studies (3–5). *C. elegans* has a short life cycle with a large brood size and is particularly amenable to genetic analyses at the organismal level. Antiviral RNAi was first shown to inhibit the replication of Flock House virus (FHV) launched from an integrated transgene in the worm genome and the infection of primary worm cells with vesicular stomatitis virus (VSV) (6–8). FHV is a member of the *Nodaviridae*, which include viruses with a bipartite positive-strand RNA genome. VSV contains a nonsegmented negative-strand RNA genome. However, neither FHV nor VSV naturally infects *C. elegans*. Orsay virus (OrV), the first virus known to naturally infect *C. elegans* in the wild, was reported in 2011 by a collaborative team among the Felix, Miska, and Wang laboratories (9). OrV and the related Le Blanc and Santeuil viruses that infect *Caenorhabditis briggsae* all contain a closely related bipartite positive-strand RNA genome most similar to that of nodaviruses (9–13). Notably, genetic studies have shown that FHV, VSV, and OrV are all targeted in *C. elegans* by a conserved antiviral RNAi pathway (9, 14–18) mechanistically similar to exogenous RNAi triggered by long dsRNA, characterized extensively since its discovery in 1998 (19).

In exogenous RNAi, long dsRNA is processed into siRNAs by the single nematode Dicer in complex with dsRNA-binding protein RDE-4 (20, 21). These Dicer-dependent primary siRNAs are predominantly 23 nt long and do not show nucleotide preferences at the 5′ termini. After loading into RISC containing Argonaute RDE-1, these primary siRNAs recognize the target RNA via base-pairing and recruit cellular RNA-dependent RNA polymerase (RdRP) RRF-1 in complex with Dicer-related helicase 3 (DRH-3) to synthesize antisense secondary siRNAs in somatic cells (22–24). Known also as 22G siRNAs, mature secondary siRNAs are predominantly 22 nt long, contain triphosphates at the 5′ ends, overwhelmingly prefer guanosine as the 5′-terminal nucleotide, are much more abundant than the primary siRNAs, and are loaded into worm-specific Argonaute proteins to guide target RNA clearance (23, 25). Secondary siRNAs also play an essential role in RNAi in plants, although they are the products of Dicer and thus have the same biochemical properties as the primary siRNAs (26, 27). Antiviral RNAi triggered by OrV infection in *C. elegans* is also associated with the production of both the primary and secondary vsiRNAs (14, 17). Defective antiviral RNAi in *rde-1*, *rrf-1*, and *drh-3* mutant worms is correlated with the loss of the viral 22G siRNAs even though the 23-nt primary vsiRNAs processed by Dicer from viral dsRNA replicative intermediates are highly abundant (14, 17). Consistently, FHV RNA replication induces accumulation of highly abundant 23-nt primary vsiRNAs in *rde-1* mutant worms (28, 29). Similarly to exogenous RNAi, therefore, 22G siRNAs are essential for antiviral RNAi.

Intriguingly, Dicer-related helicase 1 (DRH-1) is essential for antiviral RNAi but is largely dispensable for exogenous RNAi (7, 14, 17), revealing a specific genetic requirement for antiviral RNAi. DRH-1 and DRH-3 are highly homologous to the family of mammalian RIG-I-like receptors (RLRs) (20), which act as intracellular viral RNA sensors to initiate the interferon-regulated innate immunity against RNA virus infection in mammals (30, 31). RLRs, which are found in neither fruit flies nor plants, include three members in mammals: retinoic acid-inducible gene I (RIG-I), melanoma differentiation-associated protein 5 (MDA5), and Laboratory of Genetics and Physiology 2 (LGP2). The central helicase domain and the C-terminal regulatory domain of RLRs are required for the sensing of specific intracellular viral RNAs, including viral dsRNA (30, 31). RIG-I and MDA5, but not LGP2, contain an N-terminal tandem caspase activation and recruitment domain (CARD) necessary for downstream innate immune signaling upon the detection of the intracellular viral RNA, leading to the production of type I interferons and the expression of numerous interferon-stimulated genes. DRH-1 and DRH-3 as well as DRH-2 share sequence homology with mammalian RLRs in the central helicase and C-terminal domains (7, 20). However, the N-terminal domain conserved in DRH-1 and DRH-3 shares no sequence similarity with CARD and is absent in DRH-2. The helicase domains of RLRs and DRHs are closely related to that found in Dicer proteins encoded by diverse eukaryotes (20, 32, 33). Pyle and colleagues have recently designated these proteins duplex RNA-activated ATPases since their helicase/ATPase domains contain a unique α-helical insertion domain (Hel2i) and lack dsRNA unwinding activity of the RNA helicases (34). It is known that RIG-I translocates along dsRNA during the activation of innate immune signaling in a process powered by ATP hydrolysis (35).

DRH-1 was initially identified as a component in the complex with both Dicer and RDE-4 (20). The antiviral role of DRH-1 was discovered because FHV replicated to significantly enhanced levels after depletion of *drh-1* mRNA or in worm mutants homozygous for a loss-of-function *drh-1* allele (7). A natural antiviral function of DRH-1 was recently illustrated elegantly by the identification of a 159-bp deletion polymorphism in the *drh-1* gene of wild *C. elegans* isolates exhibiting defective antiviral RNAi against OrV infection using a genome-wide association study (14). The accumulation of FHV-specific vsiRNAs in *drh-1* mutant worms is readily detectable by Northern blot hybridization (7, 17). Notably, efficient silencing of cellular genes targeted in *trans* by complementary vsiRNAs induced by FHV replication is abolished in *rde-1* and *rrf-1* mutants but not in *drh-1* mutant worms (15, 17). Thus, *drh-1* mutant worms produce functional secondary vsiRNAs possibly at reduced levels that are sufficient to inhibit the expression of cellular genes but not the replication of viral genomic RNAs. These findings suggest that DRH-1 acts downstream of the biogenesis of vsiRNAs in antiviral RNAi (7, 17).

A recent study has comprehensively characterized the populations of the primary and secondary vsiRNAs produced by a panel of wild-type and mutant *C. elegans* strains after infection with OrV (14). Consistent with the previous study (7), *drh-1* mutants display no difference in endogenous 22G siRNAs mapping antisense to protein-encoding genes (14). OrV-infected *drh-1* mutant worms indeed accumulate low levels of the secondary vsiRNAs (14), which appears to explain why cellular genes are silenced by vsiRNAs in *drh-1* mutant worms (17). However, the size distribution of the total virus-derived small RNAs mapped to the RNA genome of OrV suggested a severe defect in the biogenesis of the 23-nt primary vsiRNAs in *drh-1* mutant worms (14). Interestingly, both the ATPase and C-terminal domains of human RIG-I can substitute for the corresponding domains of DRH-1 to mediate antiviral RNAi in *C. elegans* (17). Moreover, the KWK motif conserved in the C-terminal domains of RIG-I and DRH-1 is required for antiviral RNAi in *C. elegans* (17). These findings suggest a new model for DRH-1 function in which, similarly to mammalian RLRs, DRH-1 acts as an intracellular dsRNA sensor to initiate antiviral RNAi in *C. elegans* (14).

In this study, we developed an organism-level, unbiased screen for the genetic dissection of the antiviral RNAi pathway in *C. elegans*. Using chemical mutagenesis and a mapping approach based on whole-genome sequencing, we isolated and cloned four novel mutant alleles of *drh-1* located in the conserved ATPase and C-terminal domains as well as in the worm-specific N-terminal domain. Deep sequencing of small RNAs revealed accumulation of Dicer-dependent vsiRNAs targeting OrV genomic RNAs in multiple *drh-1* single and double mutant animals. Our findings show that, in contrast to mammalian RLRs, DRH-1 does not act as the receptor to initiate antiviral RNAi in *C. elegans*. We discuss a model in which a predicted dsRNA translocase activity of DRH-1 is involved in the processing of viral dsRNA replicative intermediates by the DCR-1/RDE-4/DRH-1 complex.

## RESULTS

### Identification of *C. elegans* mutants with enhanced virus susceptibility from forward genetic screens.

We constructed a *C. elegans* strain carrying a single-copy, heat-inducible transgene that directed transcription of a self-replicating FHV RNA1 derivative, FR1gfp, described previously (see Fig. S1A in the supplemental material) (7). The genomic RNA1 of FHV replicates to high levels in *C. elegans* in the absence of the genomic RNA2, which encodes the capsid protein, because of potent suppression of antiviral RNAi by the viral suppressor of RNAi (VSR) B2 protein, expressed from the subgenomic RNA3 produced after RNA1 replication (6). The absence of B2 expression from FR1gfp renders this RNA1 derivative highly susceptible to antiviral RNAi, which effectively inhibits the expression of enhanced green fluorescent protein (GFP) in place of B2 (7).

10.1128/mBio.00264-17.2 FIG S1  Characterization of *irSi18* (N2;*FR1gfp*) and the isolated 13 mutants defective in antiviral immunity. (A) Schematic of the FR1gfp viral replicon launched from the *irSi18* transgene. The C-terminal region of the viral RNA-dependent RNA polymerase (RdRP) is effectively truncated and replaced by the −1 reading frame of the eGFP coding sequence. Rz, hepatitis delta virus ribozyme; HIP, heat-inducible promoter. (B) Visualization of GFP expression in all 13 mutants, 48 h after heat induction of FR1gfp. (C) Northern blot detection of the accumulation of FR1gfp RNA1 and RNA3 in mutants proficient in RNAi. Download FIG S1, TIF file, 4.2 MB. Copyright © 2017 Coffman et al. 2017 Coffman et al. This content is distributed under the terms of the Creative Commons Attribution 4.0 International license.

**FIG 1:**
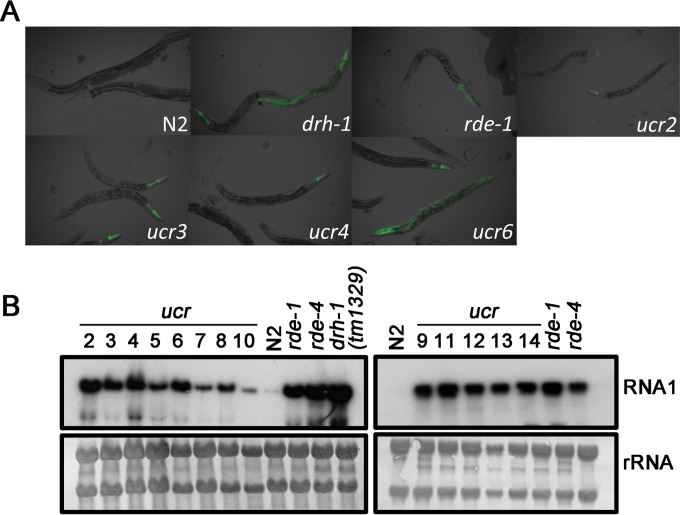
Isolation of mutants defective in antiviral immunity. (A) Synchronized L1 wild-type (N2) and mutant animals carrying the _p_hsp-16.41::FR1gfp transgene were plated and heat induced for 4 h at 34°C at the L4 stage. GFP was visualized by fluorescence microscopy 48 h after heat induction. (B) Northern blot detection of OrV RNA1 accumulation in N2 and the isolated mutants (designated *ucr*) grouped according to their susceptibility (left) or resistance (right) to exogenous RNAi, using known mutants as controls.

As expected, none of the wild-type N2 animals exhibited any visible GFP expression after the induction of FR1gfp replication (Fig. 1A). In contrast, strong GFP expression was associated with FR1gfp replication after the viral transgene was introduced into *C. elegans* mutants defective in antiviral RNAi, either in the whole body [e.g., *drh-1*(*tm1329*)] or in the pharynx [e.g., *rde-1*(*ne219*)] (Fig. 1B). Northern blot analysis further revealed that while both RNA1 and RNA3 of FR1gfp accumulated to high levels in *rde-1*, *rde-4*, and *drh-1* mutant animals, neither viral RNA was detectable in the N2 background (Fig. S1C). As described previously for N2 worms carrying a multicopy FR1gfp transgene array (7), therefore, both the replication of the recombinant FHV RNA1 and the viral GFP expression from the single-copy FR1gfp transgene in the new worm strain were potently inhibited by antiviral RNAi, establishing the N2;*FR1gfp* worm as a model for the genetic characterization of the antiviral RNAi pathway.

We subjected N2;*FR1gfp* young adults to standard ethyl methanesulfonate (EMS)-induced mutagenesis (36) and screened approximately 284,000 haploid genomes. When the mutagenized F2 generation reached the L4 stage, FR1gfp replication was induced by heat treatment, and 48 h later, worms were screened for GFP expression. Single worms expressing GFP were transferred to individual plates to establish independent F2 mutant lines. Although most of the identified mutant animals did not survive, we isolated 13 viable mutants that reproducibly failed to suppress GFP expression from FR1gfp in subsequent generations (Fig. 1A and Fig. S1B). However, we noted that the phenotypic penetrance of GFP expression was less than 100% in all of the isolated mutants.

**FIG 2:**
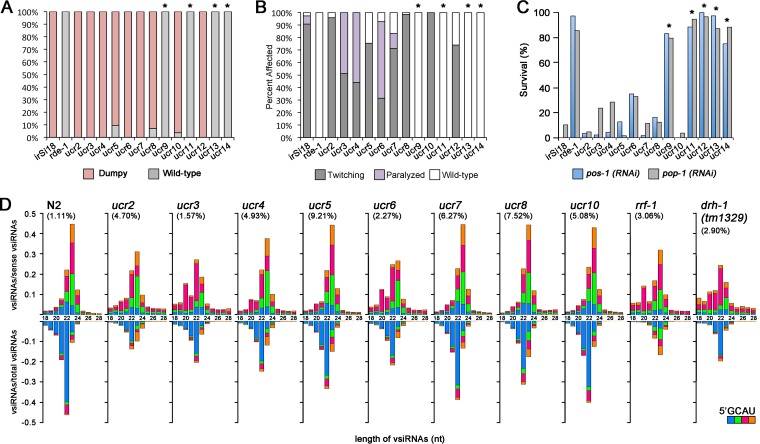
The isolated mutants are defective in exogenous and/or antiviral RNAi. (A to C) Susceptibility of the isolated mutants to exogenous RNAi to target the somatic (*dpy-7* [A] and *unc-22* [B]) and germline (*pos-1* and *pop-1* [C]) genes. Shown are the percentage of dumpy animals after RNAi of *dpy-7* (pink) or of viable animals after RNAi of *pos-1* (blue) or *pop-1* (gray), respectively. RNAi of *unc-22* (B) caused twitching (gray) or paralysis (purple). The N2;*FR1gfp* (*irSi18*) worm strain was used as the control. Mutants exhibiting similar patterns as *rde-1* worms are marked with asterisks. (D) Size distribution, polarity, and the 5′-terminal nucleotide of virus-derived small RNAs sequenced by the 5′-P-independent protocol from N2 and mutant worms 5 days after infection with Orsay virus. The relative abundance of different-size sense vsiRNAs (top) is shown as the proportion of sense vsiRNAs, whereas that of antisense vsiRNAs (bottom) is presented as the proportion of total vsiRNAs. The abundance of the total 21- to 24-nt vsiRNAs in each sequenced small RNA library is given (in parentheses).

We first determined if the isolated worm mutants were defective either in antiviral defense against OrV infection or in experimentally induced RNAi. N2 animals are resistant to OrV (5), so that the accumulation of OrV RNAs is not readily detectable by Northern blotting (Fig. 1B). We found that all of the 13 mutants were more susceptible to OrV since they reproducibly supported high-level replication of OrV (Fig. 1B). Interestingly, five of the 13 mutants, the *ucr9*, *ucr11*, *ucr12*, *ucr13*, and *ucr14* mutants, became resistant to exogenous RNAi against both genes (*pop*-*1* and *pos-1*) expressed mainly in the germline (Fig. 2C), suggesting defective antiviral RNAi in these mutants. Resistance to exogenous RNAi targeting two somatic genes, *dpy-7* and *unc-22*, was also detected in these RNAi-defective mutants, except the *ucr12* mutant (Fig. 2A and B). However, the remaining eight mutants, the *ucr2* to *ucr8* and *ucr10* mutants, supported efficient exogenous RNAi targeting the somatic and germline genes (Fig. 2A, B, and C), suggesting that the core RNAi machinery to respond to exogenous long dsRNA was not altered in these mutants. We also verified that all of these eight mutants supported replication of the recombinant FHV RNA1 and transcription of its subgenomic RNA at levels readily detectable by Northern blot hybridization (Fig. S1C).

We further sequenced the total small RNAs of the eight mutants proficient in exogenous RNAi after OrV infection to determine if any of these mutants exhibited defects in the production of the primary or secondary viral siRNAs (vsiRNAs). The small RNAs captured by the 5′-P-independent protocol include both the primary and secondary vsiRNAs containing 5′ mono- and triphosphates, respectively (14). As controls, we also sequenced the small RNAs from wild-type N2 and *rrf-1* mutant worms infected with OrV, and the properties of the vsiRNA reads mapped to the genome of OrV (11) are presented in Fig. 2D according to the format used by Miska and colleagues (14). The antisense vsiRNAs produced by N2 worms exhibited the known properties of secondary siRNAs with strong preference for both 5′-terminal guanosine (blue bars) and 22 nt, whereas the sense vsiRNAs were enriched for 23 nt without a clear 5′-terminal nucleotide preference (Fig. 2D). In contrast, 23-nt RNAs were the most abundant for both the sense and antisense vsiRNAs in *rrf-1* mutant worms, and neither the antisense nor sense vsiRNAs showed a preference for 5′-terminal guanosine (Fig. 2D). Consistent with the published data (14), therefore, deep sequencing of the total small RNAs cloned by the 5′-P-independent protocol revealed production of a typical population of the primary vsiRNAs in *rrf-1* mutant worms and of a mixed population of the primary and secondary vsiRNAs in N2 worms. We found that all of the eight mutants produced vsiRNAs in the size range of 21 to 24 nt with the relative abundance of vsiRNAs in these mutants (1.57% to 9.21%) being higher than that in N2 worms (1.11%) (Fig. 2D). However, the prevalence of both the 23-nt primary vsiRNAs and the 22G antisense secondary vsiRNAs was markedly reduced in *ucr2*, *ucr3*, *ucr4*, and *ucr6* mutants compared to N2 worms or the remaining mutants (Fig. 2D), suggesting specific defects in antiviral RNAi. These results together indicate the feasibility of discovering the genes involved in both exogenous and antiviral RNAi by the FHV replicon-based genetic screen.

### Identification of four novel alleles of *drh-1* by a mapping strategy based on whole-genome resequencing.

We mapped the causal mutations in *ucr2*, *ucr3*, *ucr4*, and *ucr6* mutants defective in antiviral RNAi but proficient in exo-RNAi by a mapping-by-sequencing strategy (see Text S1 in the supplemental material). Unexpectedly, defective antiviral RNAi in all of the four mutants was caused by a single nucleotide polymorphism (SNP) in *drh-1* and restored by transgenic expression of wild-type DRH-1 protein (see Fig. S2, S3, and S4 in the supplemental material). Specifically, whereas the *drh-1*(*ucr3*) missense allele (A72T) was located in the worm-specific N-terminal domain, the *drh-1*(*ucr4*) missense allele altered the proline residue (Pro^966^) in the C-terminal domain that is conserved in DRH-3 and mammalian RIG-I/MDA5. The *drh-1*(*ucr6*) nonsense mutation results in a premature translational termination of DRH-1 to remove the entire C-terminal domain. Finally, the *drh-1*(*ucr2*) lesion led to the use of an alternative intron splice site, resulting in an in-frame deletion of 30 amino acids in the Hel2i domain found only in the group of the dsRNA-activated ATPases.

10.1128/mBio.00264-17.1 TEXT S1  Supplemental materials and methods. Download TEXT S1, DOCX file, 0.03 MB. Copyright © 2017 Coffman et al. 2017 Coffman et al. This content is distributed under the terms of the Creative Commons Attribution 4.0 International license.

10.1128/mBio.00264-17.3 FIG S2  Schematic of the mapping strategy used to identify the causal, EMS-induced mutations from *ucr2*, *ucr3*, *ucr4*, and *ucr6*. Download FIG S2, TIF file, 0.3 MB. Copyright © 2017 Coffman et al. 2017 Coffman et al. This content is distributed under the terms of the Creative Commons Attribution 4.0 International license.

10.1128/mBio.00264-17.4 FIG S3  Identification of four loss-of-function alleles of *drh-1*. (A) Schematic of the DRH-1 gene structure with the identified *ucr* alleles indicated. The ATPase domain (divided into Hel1, Hel2i, and Hel2 motifs) and the C-terminal regulatory domain (RD) are conserved in *C. elegans* DRH-1/DRH-3 and mammalian RIG-I/MDA5. Also indicated are the regions deleted in two *drh-1* mutants characterized previously. (B) Alignments showing the region deleted by the splice site mutation in *ucr2* and the amino acid substitution at a conserved proline in *ucr4*. (C) Reverse transcription and PCR to detect a 90-nt deletion in the DRH-1 mRNA in the *drh-1*(*ucr2*) mutant compared to that in N2 animals. Download FIG S3, TIF file, 0.9 MB. Copyright © 2017 Coffman et al. 2017 Coffman et al. This content is distributed under the terms of the Creative Commons Attribution 4.0 International license.

10.1128/mBio.00264-17.5 FIG S4  Transgenic rescue of *ucr2*, *ucr3*, *ucr4*, and *ucr6*. (A) Schematic of the rescue transgene carrying wild-type DRH-1 and the coinjected marker transgene. (B) Select images overlaid with a red and green exposure from fluorescence microscopy of the rescued animals carrying the DRH-1 transgene marked by the red fluorescence from the marker mCherry (left) and the mutant animals before the injection with the rescue constructs (right). Note that strong green fluorescence from FR1gfp replication in the mutant worms (right) disappeared following the introduction of the DRH-1 transgene and they exhibited only red fluorescence (left). (C) Rescue of FR1gfp accumulation in the isolated *drh-1* mutants with a wild-type DRH-1 transgene shown by Northern blot detection of RNA1 and RNA3 from FR1gfp. Download FIG S4, TIF file, 2.6 MB. Copyright © 2017 Coffman et al. 2017 Coffman et al. This content is distributed under the terms of the Creative Commons Attribution 4.0 International license.

### Detection of abundant 23-nt vsiRNAs targeting the terminal regions of viral genomic RNAs in *drh-1* mutant worms.

Identification of multiple alleles in the *drh-1* gene from our unbiased genetic screen further supports an indispensable role of DRH-1 in antiviral RNAi in *C. elegans* revealed by previous studies (7, 14, 17). Curiously, we noted a strong peak at 23 nt for both the sense and antisense vsiRNAs in all of our four *drh-1* mutants (Fig. 2D), which was not detected previously in the *drh-1*(*ok3495*) mutant infected with OrV (14). The absence of a distinct 23-nt peak among other nonspecific sizes of OrV-derived small RNAs led to the conclusion that DRH-1 is required for the initiation of an antiviral RNAi pathway to generate the primary vsiRNAs by Dicer (14). This conclusion was in disagreement with a previous study from our group, which detected accumulation of the vsiRNAs to target the replicating FHV RNA1 in *drh-1*(*tm1329*) mutant worms by Northern blot hybridization (7). Thus, we sequenced the small RNAs cloned by the 5′-P-independent protocol from OrV-infected *drh-1*(*tm1329*) mutant worms to determine if *drh-1*(*tm1329*) mutant worms could produce vsiRNAs in response to OrV infection. As shown in Fig. 2D, the *drh-1*(*tm1329*) mutant worms produced a mixed population of the primary and secondary vsiRNAs highly similar to those detected in *ucr2*, *ucr3*, *ucr4*, and *ucr6* mutant worms.

**FIG 3:**
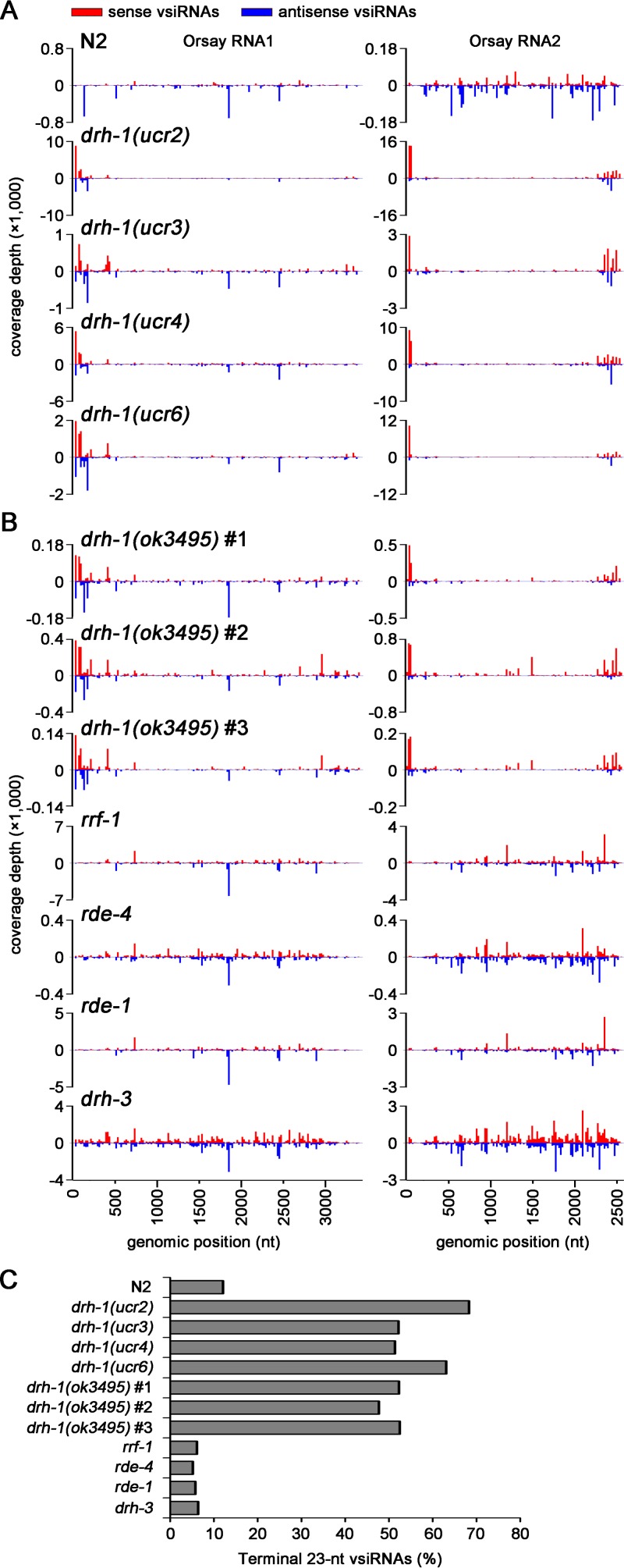
*drh-1* mutants produce highly abundant 23-nt vsiRNAs to target the terminal regions. (A and B) Mapping of 23-nt sense (red) and antisense (blue) vsiRNAs to the full-length genomic RNAs of OrV cloned from N2 and mutant animals by the 5′-P-independent protocol. The relative abundance was calculated as the 23-nt vsiRNA reads per million miRNAs. (C) Percentage of 23-nt vsiRNAs mapped to the three terminal regions (including the 3′-terminal 200-nt region of RNA2 and the 5′-terminal 200-nt regions from both RNA1 and RNA2) in N2 and mutant animals. The libraries from *drh-1*(*ok3495*), *rrf-1*, *rde-4*, *rde-1*, and *drh-3* in panels B and C were from previously published data (14).

We noted that the complete 5′- and 3′-terminal sequences of both OrV RNA1 and RNA2 (11) were not available for the initial mapping of vsiRNAs (14). Thus, the population of the 23-nt vsiRNAs that we detected in *drh-1* mutants might be derived from these terminal regions of the OrV genomic RNAs. To test this idea, we examined the distribution patterns of 23-nt vsiRNAs along the full-length genomic RNA1 and RNA2 of OrV sequenced from infected N2, *drh-1*(*ucr2*), *drh-1*(*ucr3*), *drh-1*(*ucr4*), and *drh-1*(*ucr6*) animals. Indeed, we found that in all four *drh-1* mutants, abundant 23-nt vsiRNAs in both positive and negative polarities were mapped to the 5′-terminal region of both RNA1 and RNA2 and to the 3′-terminal region of only RNA2 (Fig. 3A), which is 847 nt shorter than RNA1 (11).

We next analyzed the published libraries of vsiRNAs cloned similarly using the 5′-P-independent protocol by Miska and colleagues from a panel of worm mutants after OrV infection (14). In all of the three independent small RNA libraries from the infected *drh-1*(*ok3495*) mutant animals, high densities of the 23-nt sense and antisense vsiRNAs were found to target the 5′-terminal region of both OrV RNA1 and RNA2 and the 3′-terminal region of RNA2 (Fig. 3B). In contrast, 23-nt vsiRNA hot spots were randomly distributed across the terminal and internal regions of the two viral genomic RNAs in *rde-1*, *rde-4*, *rrf-1*, and *drh-3* mutant worms, all of which support high levels of OrV replication because of defective antiviral RNAi, similarly to *drh-1* mutant worms (7, 9, 14, 16, 17, 37).

We further divided the 23-nt vsiRNAs in each library into the terminal and internal vsiRNAs according to whether or not they are mapped to the 5′-terminal 200-nt region of both OrV RNA1 (3,421 nt) and RNA2 (2,574 nt) and the 3′-terminal 200-nt region of RNA2 (11). We found that approximately 50% of the total 23-nt vsiRNAs in the seven independent libraries cloned from distinct *drh-1* mutants were terminal vsiRNAs (Fig. 3C). In contrast, less than 12% of the total 23-nt vsiRNAs targeted the terminal regions of OrV RNAs in N2 or *rde-1*, *rde-4*, *rrf-1*, or *drh-3* mutant animals (Fig. 3C). These findings showed that distinct *drh-1* mutants accumulated abundant 23-nt vsiRNAs in response to OrV infection. Together with our previous report (7), our results demonstrate that DRH-1 is not essential for the biogenesis of the vsiRNAs induced *in vivo* by either FHV replication or OrV infection.

### The terminal vsiRNAs made by *drh-1* mutant worms exhibit the signatures of Dicer products.

**FIG 4:**
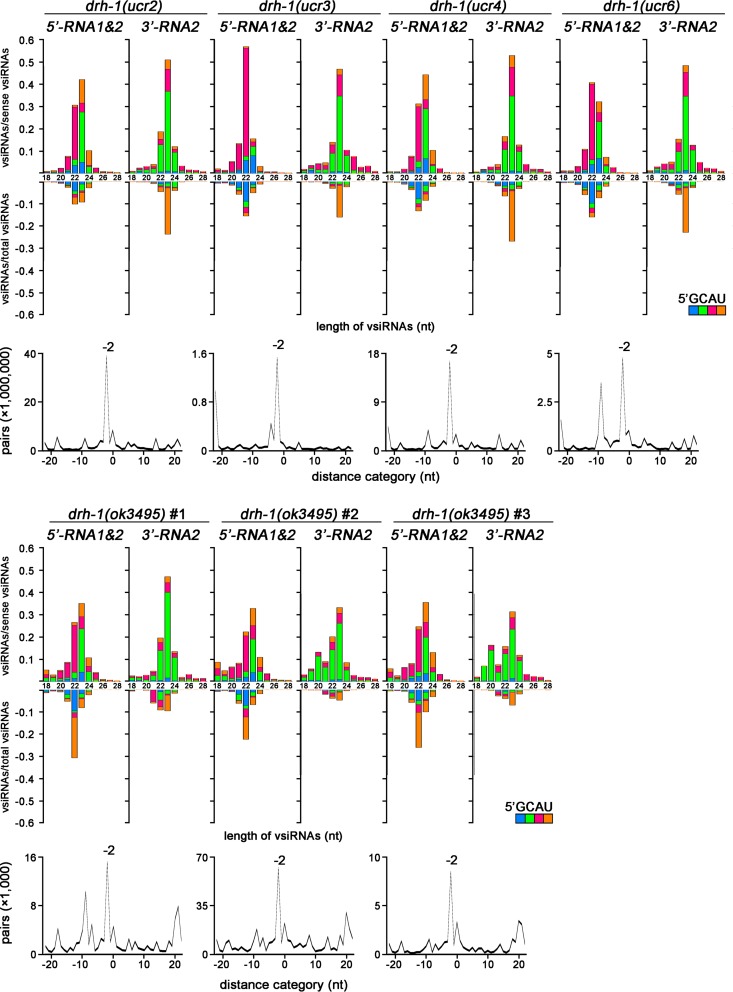
The terminal vsiRNAs cloned by the 5′-P-independent protocol from *drh-1* mutants exhibit the signatures of Dicer products. Size distribution, polarity, and the 5′-terminal nucleotide of the virus-derived small RNAs from OrV-infected *drh-1* mutant worms mapped to the 5′-terminal 200-nt regions of both RNA1 and RNA2 (top, left) or to the 3′-terminal 200-nt region of RNA2 (top, right). Computing the total pairs of the 23-nt virus small RNAs mapped to the three terminal regions with different lengths of base pairing as described previously (41) revealed strong enrichment of canonical vsiRNA pairs (−2 peak) with 2-nt 3′ overhangs (bottom). The libraries from *drh-1*(*ok3495*) were published previously (14).

We further analyzed the properties of the terminal vsiRNAs in the seven small RNA libraries cloned by the 5′-P-independent protocol from five distinct *drh-1* mutants after OrV infection. First, we examined the size distribution and the 5′-terminal nucleotide of the total 18- to 28-nt sense and antisense small RNAs mapped to the 3′-terminal 200-nt region of OrV RNA 2 and the 5′-terminal 200-nt region of OrV RNA1 and RNA2. The results showed that both of the 5′- and 3′-terminal viral small RNAs from all of the libraries exhibited a strong preference for 22 to 24 nt in the size range of Dicer products (Fig. 4, top panel in each library). Moreover, the terminal viral 23-nt RNAs were highly enriched for a 21-nt perfectly base-paired duplex with 2-nt 3′ overhangs (Fig. 4, bottom panel in each library). These findings indicate that the terminal viral small RNAs made by *drh-1* mutant worms are the primary vsiRNAs processed by Dicer from the terminal viral dsRNA replicative intermediates. Consistently, the 22- to 24-nt antisense vsiRNAs targeting the terminal regions did not show a preference for 5′-terminal guanosine in all libraries, indicating that these terminal vsiRNAs are not the secondary vsiRNAs. Interestingly, several of the terminal antisense vsiRNA populations exhibited modest enrichments for 5′-terminal uracil (Fig. 4, top panel in each library), which was similar to worm microRNAs (miRNAs) and to the influenza A virus vsiRNAs produced by human Dicer in somatic cells (38).

**FIG 5:**
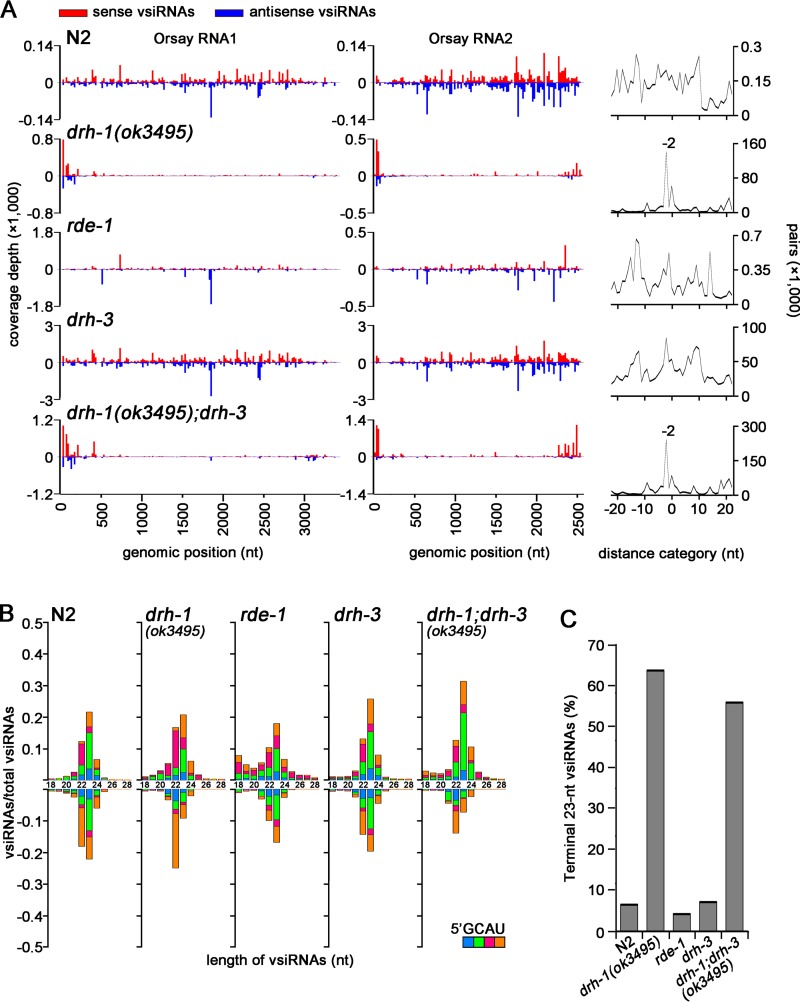
The terminal vsiRNAs cloned by the 5′-P-dependent protocol from *drh-1* mutants exhibit the signatures of Dicer products. (A) Mapping of the 23-nt sense (red) and antisense (blue) vsiRNAs cloned from N2 and mutant animals by Ashe et al. (14) to the genomic RNAs of OrV (left panel) and the enrichment of the 23-nt vsiRNAs mapped to the three terminal regions for pairs of canonical vsiRNAs with 2-nt 3′ overhangs (−2 peak, right panel). The relative abundance was calculated as the vsiRNA reads per million miRNAs. (B) Size distribution, polarity, and the 5′-terminal nucleotide of total vsiRNAs from the same libraries shown in panel A mapped to the 3′-terminal 200-nt region of RNA2 and to the 5′-terminal 200-nt regions of both RNA1 and RNA2. The relative abundance of the different-size sense (top) and antisense (bottom) vsiRNAs is shown as the proportion of total vsiRNAs. (C) Percentage of the total 23-nt vsiRNAs mapped to the three terminal regions of OrV RNA1 and RNA2 from the same libraries shown in panel A.

Dicer-dependent miRNAs and primary siRNAs contain 5′-monophosphates and are selectively captured by the 5′-P-dependent protocol (39). Thus, we analyzed the published small RNA libraries cloned from OrV-infected N2 and *drh-1*(*ok3495*), *rde-1*, *drh-3*, and *drh-1*(*ok3495*); *drh-3* mutant animals by the 5′-P-dependent protocol (14). We found that the terminal vsiRNAs did not accumulate to higher densities than the internal vsiRNAs in N2 or *rde-1* or *drh-3* mutant animals (Fig. 5A and C). In contrast, high densities of 23-nt vsiRNAs were mapped to the 5′-terminal regions of OrV RNA1 and RNA2 as well as to the 3′-terminal region of OrV RNA2 in both *drh-1*(*ok3495*) and *drh-1*(*ok3495*); *drh-3* animals (Fig. 5A). Thus, the same three terminal regions of the viral genomic RNAs were preferentially targeted by high densities of 23-nt vsiRNAs as found for the 23-nt vsiRNAs cloned by the 5′-P-independent protocol (Fig. 3A). Specifically, more than 50% of the total 23-nt vsiRNAs were mapped to these terminal 200-nt regions of the viral genomic RNAs (Fig. 5C). We found that the total sense and antisense small RNAs mapped to these terminal regions of OrV RNA1 and RNA2 exhibited a strong preference for 22 to 24 nt in the size range of Dicer products in both the single and double mutants (Fig. 5B). Moreover, the 23-nt terminal vsiRNAs were highly enriched for the 21-nt perfectly base-paired duplex with 2-nt 3′ overhangs (Fig. 5A, right panel). Therefore, examining the total small RNAs captured by the 5′-P-dependent protocol also revealed the production of Dicer-dependent vsiRNAs in *drh-1*(*ok3495*) animals after OrV infection.

Detection of Dicer-dependent biogenesis of vsiRNAs in *drh-1* mutant animals indicates that DRH-1 mediates antiviral RNAi at a step after the host immune detection and the subsequent Dicer-mediated processing of the viral dsRNA replicative intermediates. Notably, the abrupt decrease in the density of the primary vsiRNAs from the terminal regions to the internal regions of the viral genomic RNAs was observed only in *drh-1* mutant worms, not in N2 or *rde-1*, *rde-4*, or *drh-3* mutant worms (Fig. 3 and 5). Thus, the presence of DRH-1 in the wild-type and mutant worms appeared to reduce the production of the terminal vsiRNAs and/or enhance Dicer-dependent production of the primary vsiRNAs to target the internal regions of the viral genomic RNAs.

### Genetic requirements for the biogenesis of the DRH-1-independent terminal vsiRNAs.

**FIG 6:**
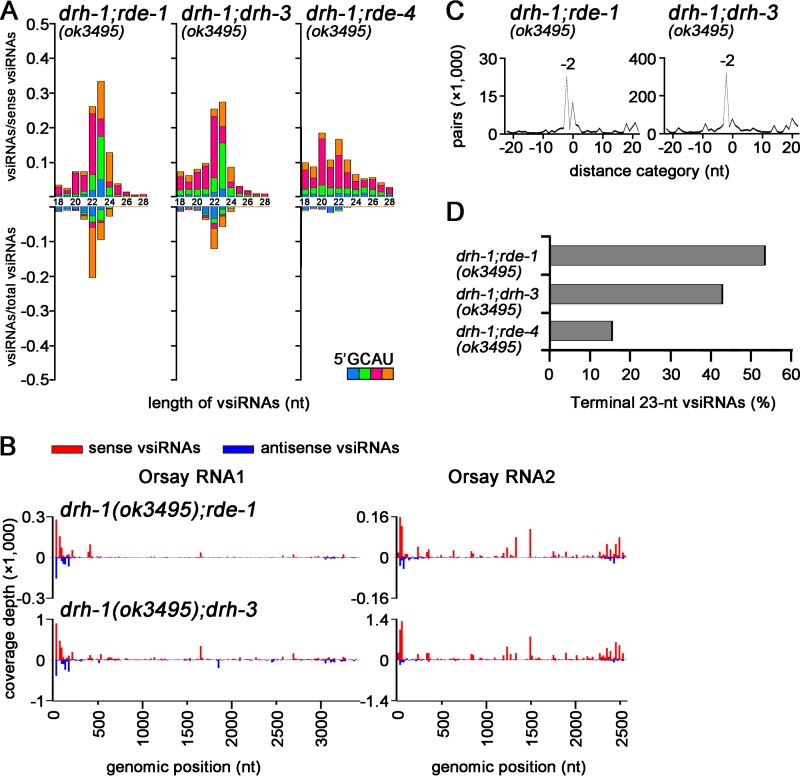
Genetic requirements for biogenesis of the *drh-1*-independent vsiRNAs. (A) Size distribution, polarity, and the 5′-terminal nucleotide of total small RNAs cloned by the 5′-P-independent protocol by Ashe et al. (14) that were mapped to the 3′-terminal 200-nt region of RNA2 and the 5′-terminal 200-nt regions from both RNA1 and RNA2. (B) Mapping of the 23-nt sense (red) and antisense (blue) vsiRNAs to the genomic RNAs of OrV obtained from two double mutant animals from the same libraries shown in panel A. (C) Strong enrichment of the 23-nt vsiRNAs from the three terminal regions for pairs (−2 peak) of canonical vsiRNAs with 2-nt 3′ overhangs in the two double mutant worms by computing total pairs of 23-nt vsiRNAs with different lengths of base pairing as described previously (41). (D) Percentage of the total 23-nt vsiRNAs mapped to the three terminal regions from the same libraries shown in panel A.

We further analyzed the total small RNAs mapped to the complete genome of OrV in the total small RNA libraries cloned by the 5′-P-independent protocol from OrV-infected double mutants that combine *drh-1*(*ok3495*) with *rde-1*, *rde-4*, or *drh-3* (14). We found that viral small RNAs mapped to the 5′- and 3′-terminal regions of the OrV genome exhibited a strong preference for 22 and 23 nt in *drh-1*; *rde-1* and *drh-1*; *drh-3* double mutants (Fig. 6A), similarly to those cloned from each individual single mutant (Fig. 4; see also Fig. S5 in the supplemental material). High densities of the 23-nt vsiRNAs from both of the worm double mutants were mapped to the 5′-terminal region of OrV RNA1 and RNA2 as well as to the 3′-terminal region of RNA2 (Fig. 6B). Moreover, the terminal 23-nt vsiRNAs were highly enriched for the 21-nt perfectly base-paired duplex with 2-nt 3′ overhangs (Fig. 6C), the density of the terminal vsiRNAs was clearly higher than that of the internal vsiRNAs (Fig. 6D), and the antisense vsiRNAs targeting the terminal regions did not exhibit a preference for 5′-terminal guanosine (Fig. 6A) in these two worm mutants. Thus, *drh-1*; *rde-1* and *drh-1*; *drh-3* mutants produced a population of the terminal primary vsiRNAs indistinguishable from those detected in distinct *drh-1* single mutants in response to OrV infection, indicating that neither RDE-1 nor DRH-3 may play a role in the biogenesis of the terminal primary vsiRNAs.

10.1128/mBio.00264-17.6 FIG S5  Robust production of vsiRNAs in *rde-1*, *drh-3*, and *rde-4* single mutant worms infected by OrV. Size distribution, polarity, and the 5′-terminal nucleotide of total small RNAs cloned by the 5′-P-independent protocol by Ashe et al. (14) that were mapped to the 3′-terminal 200-nt region of RNA2 and the 5′-terminal 200-nt regions from both RNA1 and RNA2. Download FIG S5, TIF file, 0.1 MB. Copyright © 2017 Coffman et al. 2017 Coffman et al. This content is distributed under the terms of the Creative Commons Attribution 4.0 International license.

In contrast, the viral small RNAs mapped to the terminal regions of the OrV genome in *drh-1*(*ok3495*); *rde-4* double mutant animals were predominantly positive strands, exhibited no preference in the size range of Dicer products (Fig. 6A), and were not more abundant than those mapped to the internal regions of the OrV genome (Fig. 6D), unlike those cloned from either *drh-1* or *rde-4* single mutant animals (Fig. 4; Fig. S5). Thus, the terminus-derived small RNAs accumulated in the *drh-1*(*ok3495*); *rde-4* double mutant were similar to those mapped to the remaining regions of the OrV genome (14) and did not exhibit properties expected for Dicer products, indicating an essential role for RDE-4 in the biogenesis of the terminal primary vsiRNAs in the absence of DRH-1. Together, our findings indicate that the terminal vsiRNAs produced in mutant animals defective in *drh-1* function are dependent on DCR-1 and RDE-4 but independent of RDE-1 and DRH-3.

## DISCUSSION

In this study, we developed a small-animal model for forward genetic screens by chemical mutagenesis to identify genes required for antiviral RNAi in *C. elegans*. We isolated 13 viable mutants that supported markedly enhanced *in vivo* accumulation of a mutant FHV replicon defective in the suppression of antiviral RNAi due to the genetic inactivation of its VSR. Notably, the isolated mutants were all more susceptible than N2 worms to the infection by OrV and included five mutants defective in RNAi induced by synthetic long dsRNA, demonstrating the potential of the FHV replicon approach to studying antiviral RNAi in *C. elegans*. We show that EMS-induced mutations can be mapped and cloned by whole-genome sequencing of pooled F2 progeny from a single backcross with the parental strain even though the phenotypic penetrance is incomplete. Notably, the causal mutation in all of the four mutants defective in antiviral RNAi but proficient in exogenous RNAi was mapped to independent single nucleotide substitutions in the *drh-1* gene, further supporting a central role of the host protein in antiviral RNAi as indicated by previous studies (7, 14, 17).

We discovered an abundant population of vsiRNAs targeting the terminal regions of OrV genome in distinct *drh-1* mutants, which escaped detection in a previous analysis possibly because the complete genome sequence of OrV was unavailable for mapping vsiRNAs at the time of the study (14). These vsiRNAs target the 5′-terminal regions of both RNA1 and RNA2 as well as the 3′-terminal region of only RNA2, contain 5′-monophosphates, exhibit a size preference in the range of Dicer products, and are highly enriched for canonical siRNA duplexes with 2-nt 3′ overhangs, indicating that they correspond to the primary vsiRNAs processed by the single worm Dicer. Examination of the published libraries of small RNAs from *drh-1* single and double mutants infected with OrV (14) indicates that the terminal vsiRNAs are RDE-4 dependent but independent of either RDE-1 or DRH-3, consistent with the known Dicer-dependent biogenesis pathway for the primary exogenous and viral siRNAs (7, 14, 17, 22–25). FHV replication in *Drosophila melanogaster* cells triggers the Dicer-2 processing of highly abundant 5′-terminal dsRNA replicative intermediates of both RNA1 and RNA2, synthesized during the initiation of the progeny positive-strand RNA replication from the 3′ end of the negative-strand RNA templates (1, 40). A similar mechanism may be responsible for the biogenesis of the 5′-terminal vsiRNAs in *C. elegans*. Detection of abundant vsiRNAs to target only the 3′-terminal region of OrV RNA2 in worms (Fig. 3A, 5A, and 6B) suggests that RNA2 is perhaps more active than RNA1 as the template for the negative-strand RNA synthesis by the viral replicase. Our findings together show that the essential function of DRH-1 in antiviral RNAi occurs at a step after the initial Dicer-dependent processing of the viral dsRNA replicative intermediates into the primary vsiRNAs. Thus, detection of the primary vsiRNAs, the immediate product after immune sensing of viral dsRNA by the RNAi machinery, allows us to conclude that DRH-1 does not act as the immune receptor of antiviral RNAi. This is in contrast to the receptor function of mammalian RLRs in innate immunity against RNA virus infections (30, 31).

Recent studies indicate that Dicer proteins, mammalian RLRs, and worm DRH-1/DRH-3 are duplex RNA-activated ATPases (34). We found that Dicer-dependent primary vsiRNAs produced in distinct *drh-1* mutant worms were mapped predominantly to the terminal regions of the viral genomic RNAs. The *drh-1* mutants examined in this work included *drh-1*(*ucr2*), *drh-1*(*ucr4*), and *drh-1*(*ucr6*) mutants that contained independent, EMS-induced mutations in the specific domains of DRH-1 that are highly conserved among the duplex RNA-activated ATPases (34). In contrast, the hot spots of the primary vsiRNAs were randomly distributed across the viral genomic RNAs in either wild-type N2 worms or RNAi-detective mutants such as *rde-1*, *rde-4*, *rrf-1*, and *drh-3* mutants. These findings suggest that DRH-1 acts to reduce the biogenesis of the terminal primary vsiRNAs and/or enhance Dicer-dependent production of the primary vsiRNAs to target the internal regions of the viral genomic RNAs in *C. elegans*. Given that RIG-I can translocate on long dsRNA powered by ATP hydrolysis (35), a similar translocase activity predicted for DRH-1 (34) would facilitate the rapid translocation of the known DCR-1/RDE-4/DRH-1 complex (20) on the viral dsRNA precursors. Presumably, this enzymatic activity of DRH-1 would allow the processing of the internal regions of the viral dsRNA into primary vsiRNAs to ensure the subsequent production of the secondary vsiRNAs at levels sufficiently high to inhibit the replication of OrV and FHV. It is known that cellular mRNAs are targeted for efficient RNAi by the vsiRNAs made in *drh-1* mutants (15, 17), indicating that the enhanced biogenesis of vsiRNAs by DRH-1 confers a specific antiviral function in *C. elegans*. Recent studies have demonstrated Dicer-dependent processing of viral dsRNA replicative intermediates into vsiRNAs in mammals (38, 41, 42). Thus, it would be interesting to determine if RLRs and interferon signaling play a role in the biogenesis of vsiRNAs targeting RNA virus infections in mammals.

## MATERIALS AND METHODS

### Worm maintenance and genetics.

*C. elegans* genetics and culture, heat induction of the recombinant RNA1 transgene of FHV (FR1gfp), and Northern blot detection of FHV RNA1 and RNA3 were as described previously (6, 7). The _p_hsp-16.41::FR1gfp transgene used to generate a single-copy insertion in this study (see Text S1 in the supplemental material) was a derivative of the _p_hsp-16.41::FR1gfp transgene previously described (6, 7) by replacing the self-cleaving ribozyme in the original construct with the ribozyme from hepatitis delta virus. The resultant worm strain, designated N2;*FR1gfp* (N2;*irSi18*), was subjected to EMS mutagenesis using standard techniques (36). FHV replication was induced at the L4 stage, and F2 worms expressing any GFP were transferred individually to their own plate and left to lay eggs. Each mutant selected was backcrossed to N2;*irSi18* at least four times, and the phenotype of the induced viral GFP expression was verified two additional times.

### Preparation of whole-genome libraries and mapping by genome resequencing.

One hundred F2s from a backcross of each mutant to the N2;*FR1gfp* parent were transferred to individual plates and given time to lay eggs. Each F2 was used in single-worm DNA extractions to be genotyped later. Once the F3s reached the L4 stage, the segregation of their viral GFP expression phenotype was scored to determine if the F2 was wild type or a heterozygous or homozygous mutant. The F3 populations from 20 homozygous mutant F2s and 20 homozygous wild-type F2s were pooled, and DNA was extracted using the Gentra Puregene kit (Qiagen). The DNA was sheared using a Bioruptor (30 s on, 30 s off, for 15 min). To construct libraries for sequencing, 1 μg from each pool was used to generate a library using the PCR-free TruSeq DNA kit (Illumina). The samples were multiplexed to have six libraries in each lane for paired-end sequencing of 100-nt reads on an Illumina 2500 sequencer. Mutant and wild-type libraries for *ucr2* were constructed using the NEXTflex PCR-free DNA library preparation kit for Illumina (Bioo Scientific), which gave lower genome coverage than the other three mutants. Mapping of the causal mutations in the four mutants by whole-genome sequencing and computational analysis was essentially as described previously (43).

### Preparation and analysis of small RNA libraries.

Infection of N2 and mutant worms with OrV was done as described previously (9, 14). Extraction of small RNAs and removal of the 5′-triphosphate groups from the secondary small RNAs by RNA 5′-polyphosphatase (RPP) were done as described previously (39). RPP-treated, purified small RNAs were used in the generation of small RNA libraries using the TruSeq small RNA sample preparation kit (Illumina). Samples were multiplexed and sequenced on an Illumina 2500 sequencer. The analysis of small RNAs was done as described previously (41). We removed the reads from each library that aligned with zero mismatches to the sense strand of structural small RNAs (25). The resultant nonstructural small RNA reads were used in the following analyses. The nonstructural small RNAs were aligned to *C. elegans* miRNAs obtained from WormBase (WS240) and used to normalize vsiRNA reads. Small RNA reads were aligned to the OrV genome (GenBank identifiers [IDs] HM030970.2 and HM030971.2), allowing zero mismatches essentially as described previously (41). For RPP-treated libraries, the sense vsiRNAs were normalized to the total sense vsiRNAs, whereas antisense reads were normalized to total vsiRNAs as described previously (14).

10.1128/mBio.00264-17.7 TABLE S1  Properties of viral small RNAs in the libraries constructed from different mutant worms infected by Orsay virus. Download TABLE S1, TIF file, 0.8 MB. Copyright © 2017 Coffman et al. 2017 Coffman et al. This content is distributed under the terms of the Creative Commons Attribution 4.0 International license.

10.1128/mBio.00264-17.8 TABLE S2  DNA libraries constructed to map the causal mutations. Download TABLE S2, TIF file, 0.4 MB. Copyright © 2017 Coffman et al. 2017 Coffman et al. This content is distributed under the terms of the Creative Commons Attribution 4.0 International license.
