# Supplementary figures and images for: Caenorhabditis elegans RIG-I Homolog Mediates Antiviral RNA Interference Downstream of Dicer-Dependent Biogenesis of Viral Small Interfering RNAs
Source: mBio. 2017 Mar 21;8(2):e00264-17. doi: 10.1128/mBio.00264-17 (PMC5362034; doi:10.1128/mBio.00264-17)

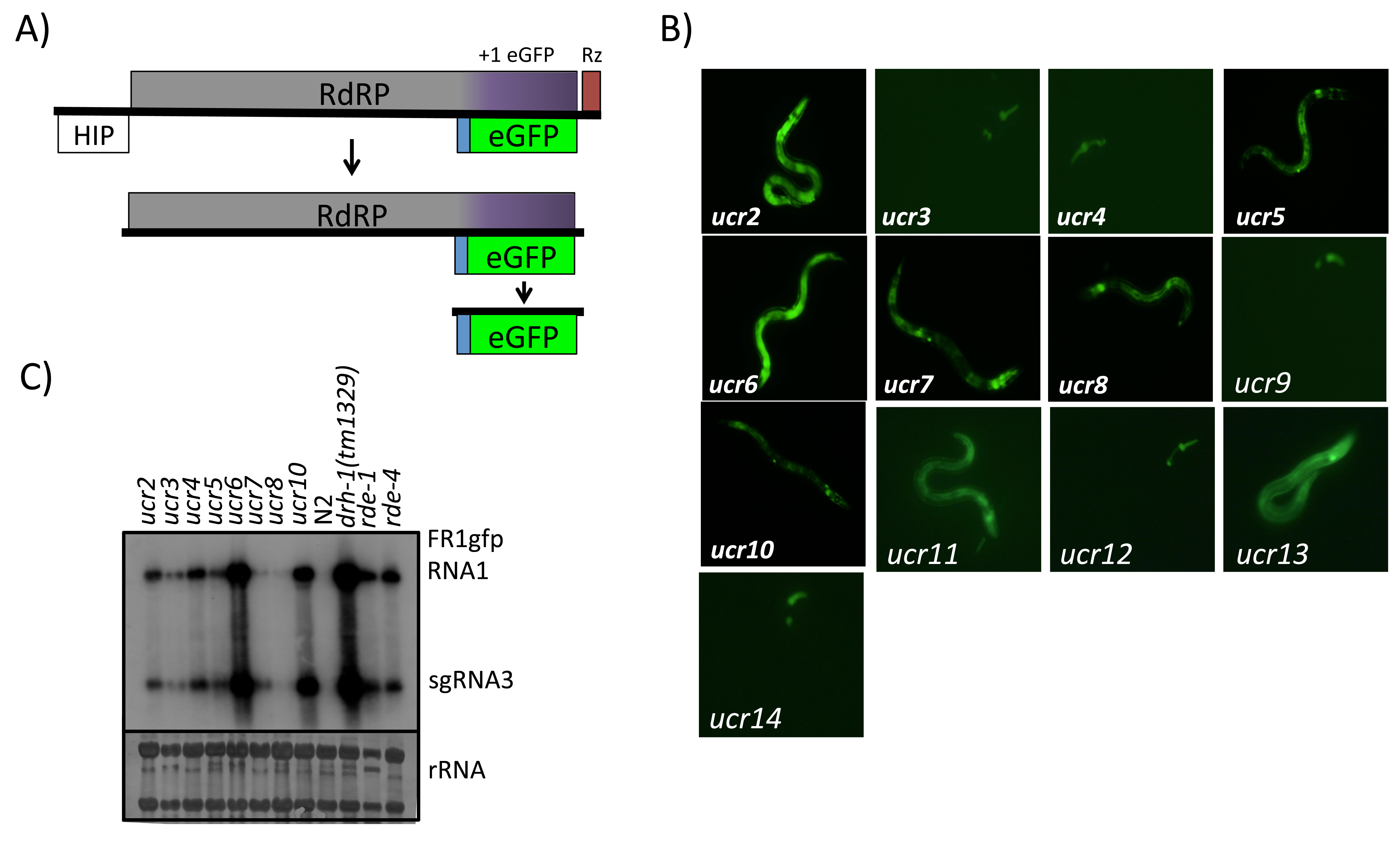

Supplement: FIG S1 [file mbo002173242sf1.tif]

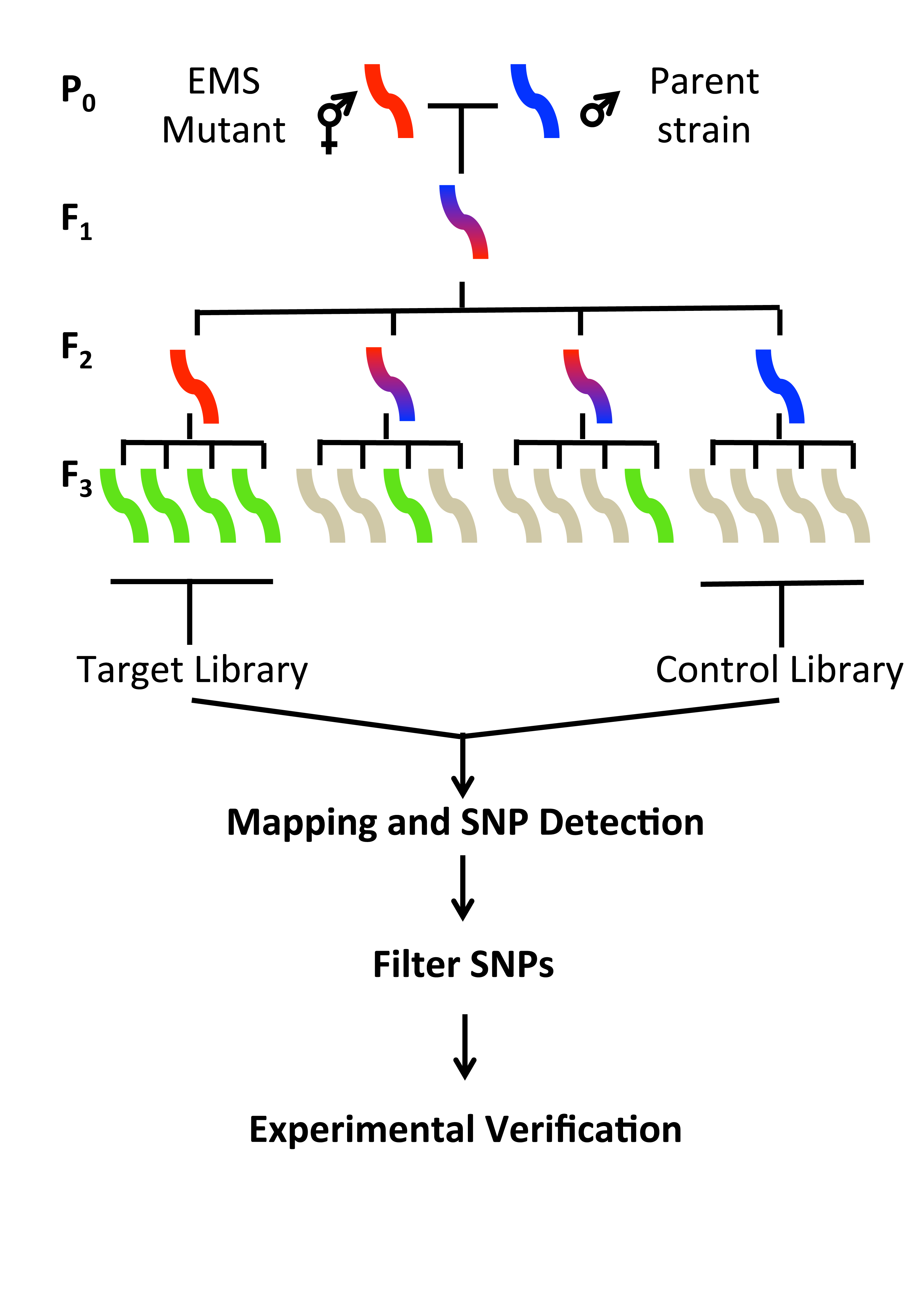

Supplement: FIG S2 [file mbo002173242sf2.tif]

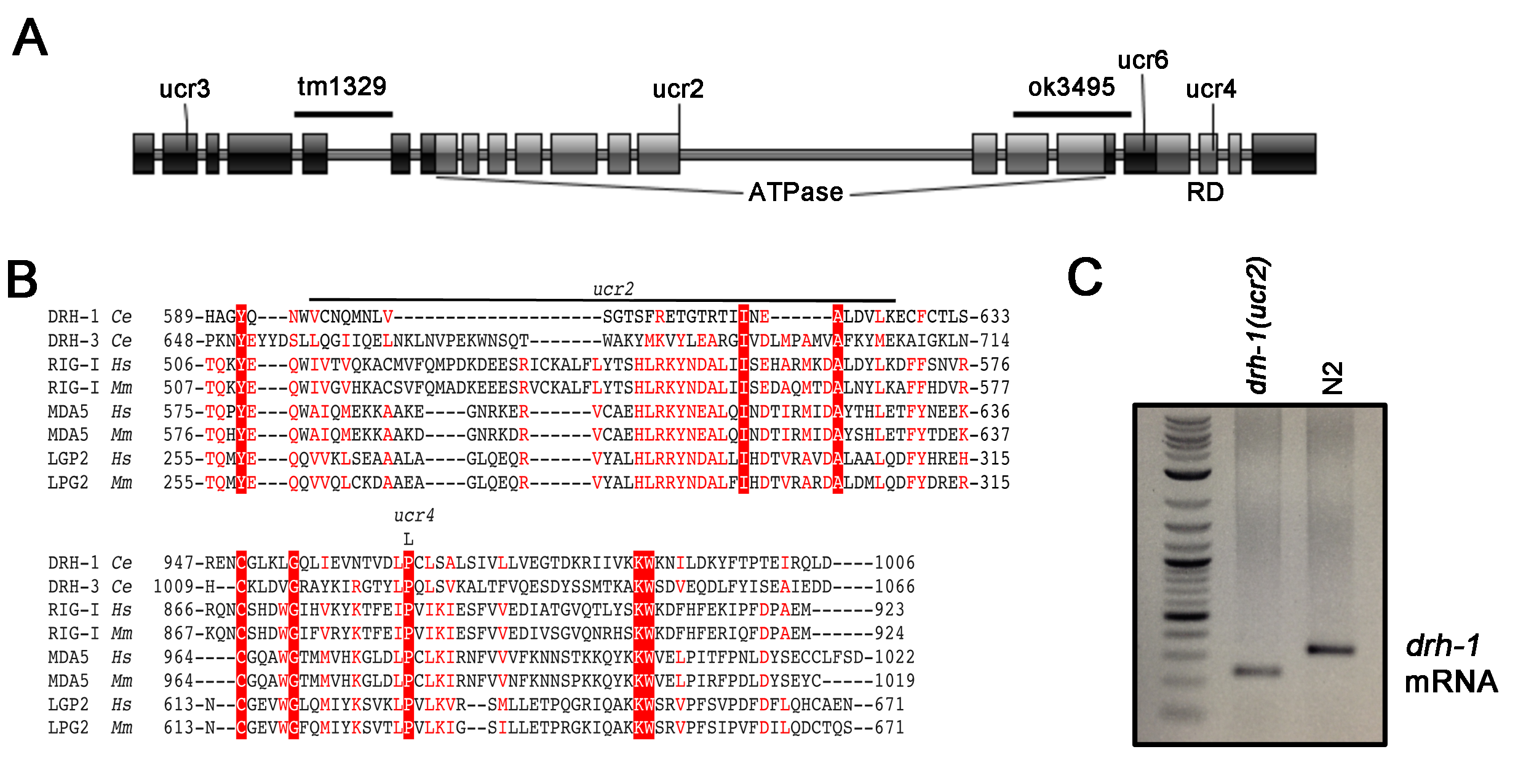

Supplement: FIG S3 [file mbo002173242sf3.tif]

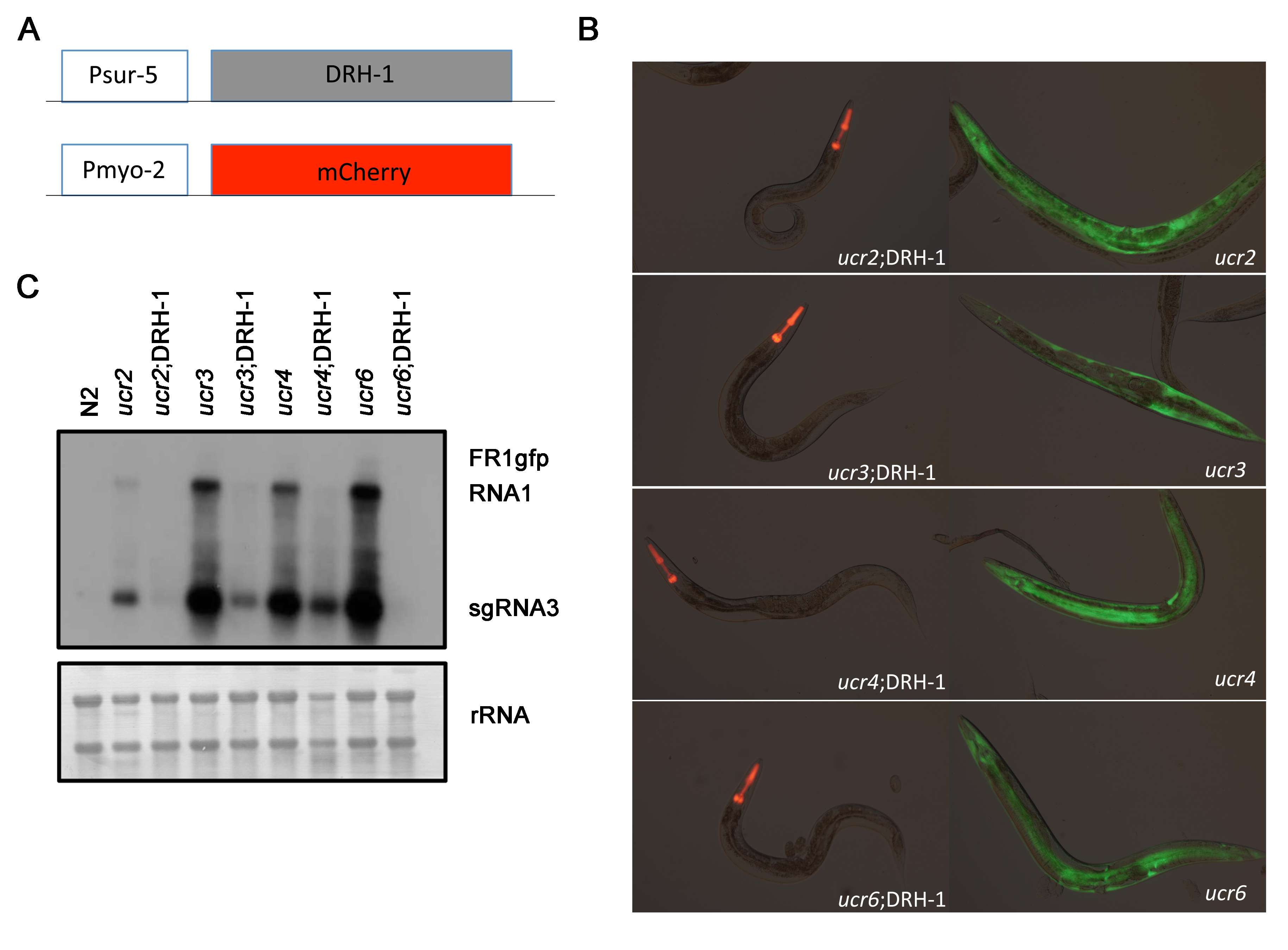

Supplement: FIG S4 [file mbo002173242sf4.tif]

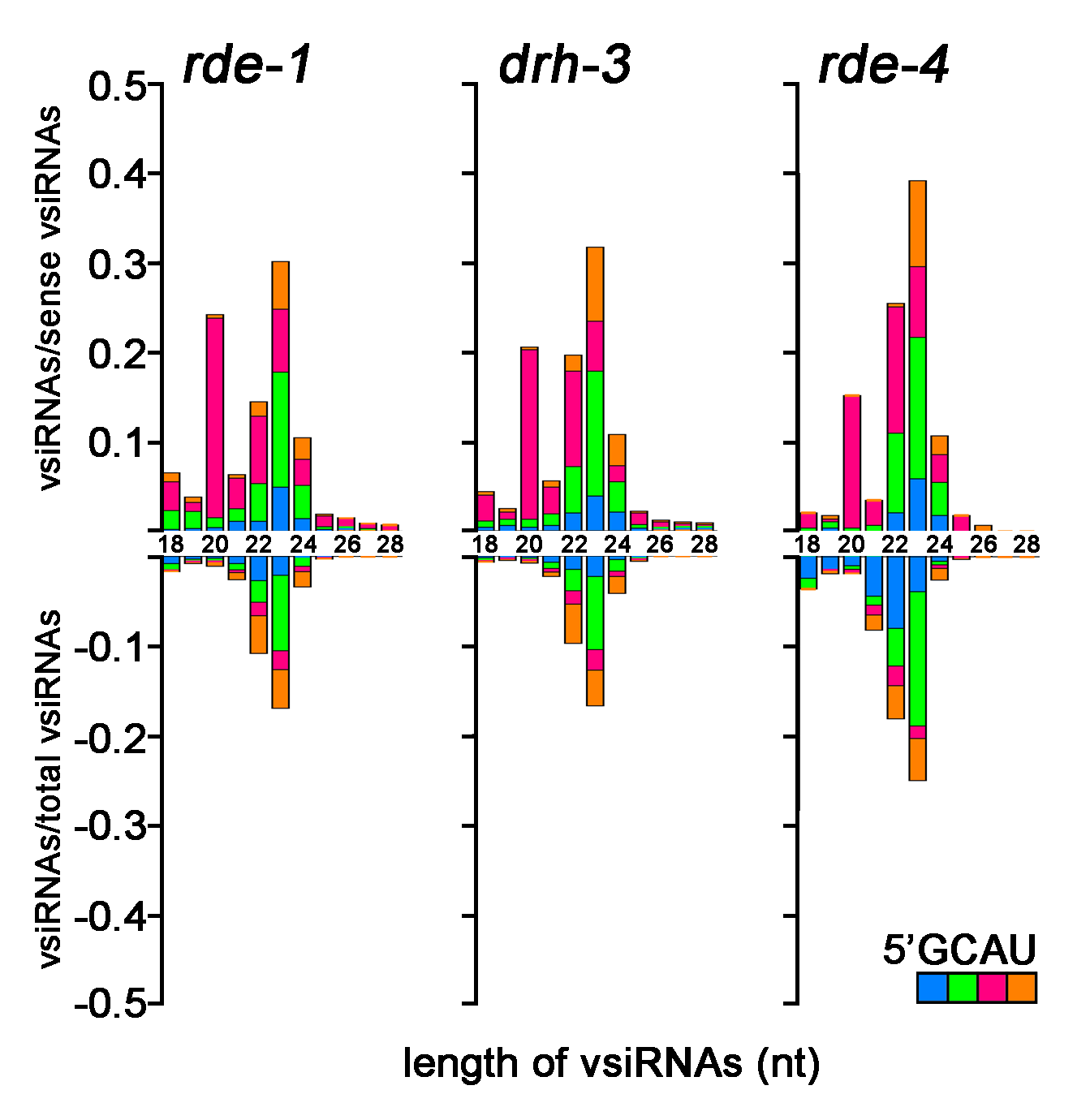

Supplement: FIG S5 [file mbo002173242sf5.tif]

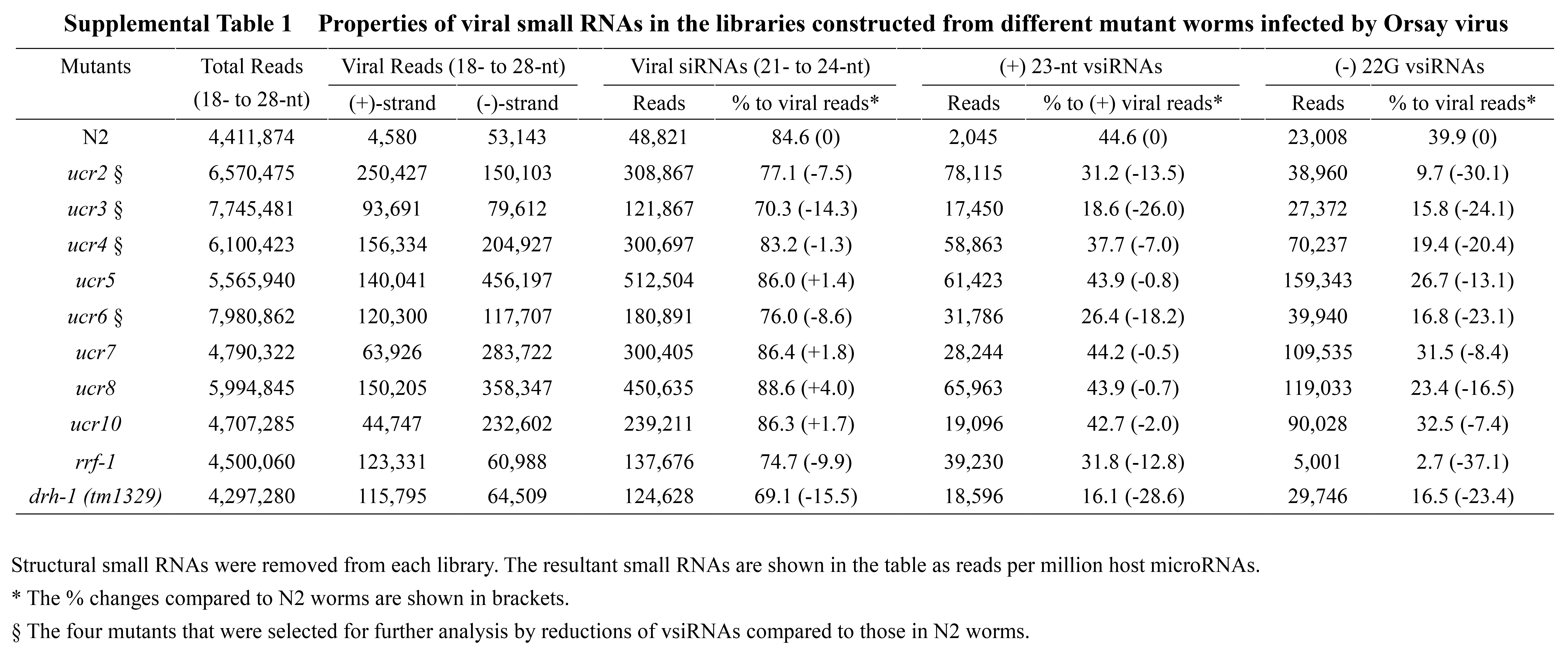

Supplement: TABLE S1 [file mbo002173242st1.tif]

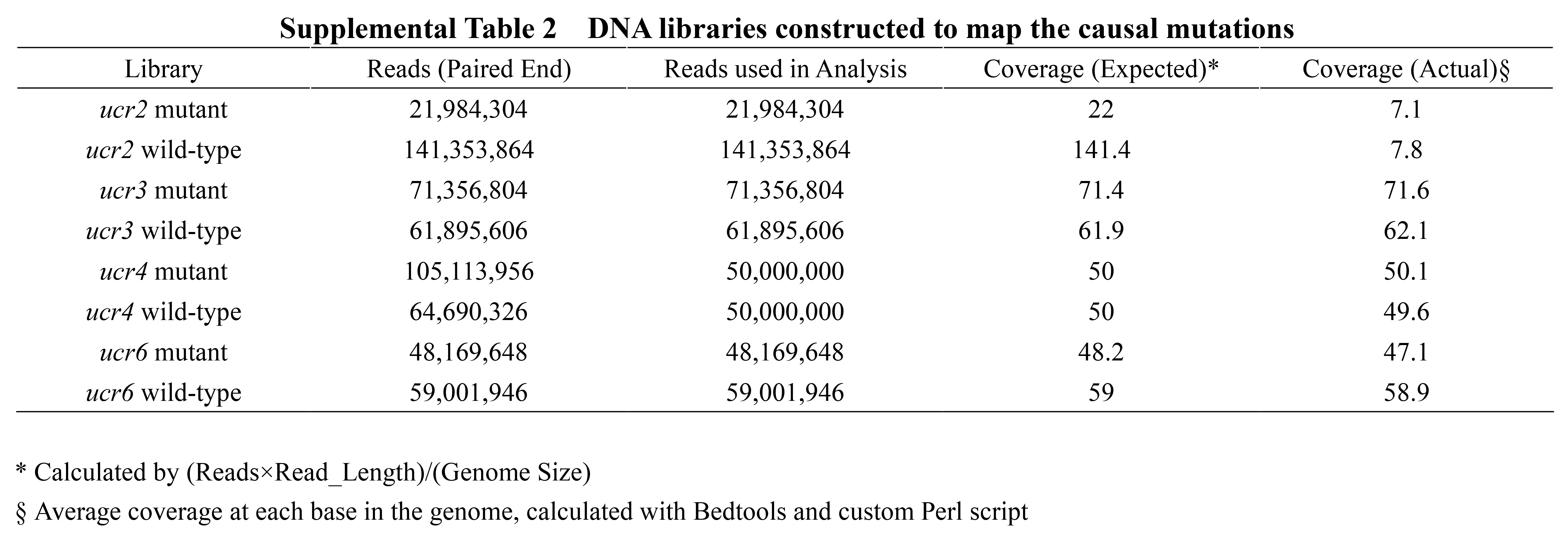

Supplement: TABLE S2 [file mbo002173242st2.tif]
