# Supplementary material for: Caenorhabditis elegans RIG-I Homolog Mediates Antiviral RNA Interference Downstream of Dicer-Dependent Biogenesis of Viral Small Interfering RNAs
Source: mBio. 2017 Mar 21;8(2):e00264-17. doi: 10.1128/mBio.00264-17 (PMC5362034; doi:10.1128/mBio.00264-17)
Supplement: TEXT S1 [file mbo002173242s1.docx]

**Text S1**

**Supplemental Materials and Methods**

**Worm maintenance and genetics.** The *C. elegans* isolate, Bristol N2, was used as the wildtype strain in this work. Worms were maintained on standard Nematode Growth Medium (NGM) at 20°C, unless otherwise stated. Mutants referred to in the text included the following alleles: *rde-1(ne219)*, *rde-4(ne301)* and *rrf-1(pk1417)*. The following pairs of primers were used for the genotyping of *drh-1(ucr6)* (ucr6 dCAPs F: 5’-gtggaagagctctcaactcagaat-3’ and ucr6 dCAPs R: 5’-tgcgctttatcggtatcttctctcaggactc-3’ to incorporate an *XhoI* site) and *drh-1(ucr4)* (ucr4 dCAPs F: 5’-ccttgtctgtgatccgggattctg-3’ and ucr4 dCAPs R: 5’-aaagaaaacccacaaagagctgatagccatg-3’ to incorporate an *NcoI* site).

**Transgene construct and transgenic worms**. The _p_hsp-16.41::FR1gfp transgene used in this study is a derivative of the _p_hsp-16.41::FR1gfp transgene previously described ([1](#_ENREF_1)). The self-cleaving ribozyme from the tobacco ringspot virus satellite RNA in the original construct was replaced with the ribozyme from hepatitis delta virus as described previously ([2](#_ENREF_2)). MosSCI ([3](#_ENREF_3)) was used to generate a single copy insertion of _p_hsp-16.41::FR1gfp, called *irSi18*. The MosSCI plasmid was injected into Strain EG4322, which contains the mosI insertion ttTi5605 on LG II and *unc-119(ed9) III* ([4](#_ENREF_4)). PCR with primers on either side of the insertion site (LHII: 5’-ctgaatatccatggcacatgcttcgtgc-3’ and RHII: 5’-agaagaccgttacgaaacagactgat-3’) and primers in the FR1gfp transgene (FR1gfp F: 5’-ccgaagtgcggtgatgatgg-3’ and FR1gfp R: 5’-tcagggtggtcacgagggtgg-3’) were used to verify the presence of the transgene on LGII. *irSi18* was crossed with *drh-1(tm1329)* and *rde-1(ne219)* mutants to verify that viral replication, detected by eGFP expression, was induced by heat shock. Northern blot detection of FHV RNA3 confirmed that FR1gfp is capable of autonomous replication from the *irSi18* transgene.

**EMS mutagenesis***.* N2;*FR1gfp* (N2;*irSi18*) young adults were treated for 4 hours in 50mM Ethyl methanesulfonate (EMS) using standard mutagenesis techniques ([5](#_ENREF_5)). After thirty minutes of recovery time, ten P_0_ animals were singled onto large (60mm) plates. The following day, each P_0_ animal was removed from the plate once they had laid about ten eggs. When the F1 animals reached adulthood and had each laid an average of twenty eggs, the F1s were removed from the plate. The resultant 2,000 F2s were heat shocked at 34°C for four hours at the L4 stage. 24 and 48 hours after transcriptional induction of FR1gfp, worms expressing any GFP were singled to their own plate and left to lay eggs. Each mutant selected was backcrossed to N2*;irSi18* at least four times and the phenotype of the induced viral GFP expression was verified two additional times.

**Preparation of whole genome libraries.** 100 F2s from a backcross of each mutant to N2;*FR1gfp* parent were transferred to individual plates and given time to lay eggs. Each F2 was used in single-worm DNA extractions to be genotyped later (see above). Once the F3s reached the L4 stage, the segregation of their viral GFP expression phenotype was scored to determine if the F2 was homozygous wild-type, heterozygous or homozygous mutant. The F3 populations from 20 homozygous mutants F2s and 20 homozygous wild-type F2s were pooled and DNA extracted using the Gentra Puregene kit (Qiagen). The DNA was sheared using a biorupter (30sec on, 30 sec off, for 15 minutes). To construct libraries for sequencing, 1 μg from each pool was used to generate a library using the PCR-free Truseq DNA kit (Illumina). The samples were multiplexed to have six libraries in each lane for pair-end sequencing of 100nt reads and sequenced on an Illumina 2500. Mutant and wildtype libraries for *ucr2* were constructed using NEXTflex™ PCR-Free DNA Library Prep Kit for Illumina (Bioo Scientific), which gave lower genome coverage than the other three mutants.

**Preparation of small RNA libraries***.* Small RNAs were extracted using 4M LiCl followed by precipitation in 2 volumes ethanol. Up to 5μg of purified small RNAs was used in a RNA 5’ Polyphosphatase (RPP) reaction to remove the 5’-triphosphate groups from the secondary small RNAs ([6](#_ENREF_6)). The reactions were incubated for 30 minutes at 37°C, per the manufacture’s method (Epicentre). RPP was removed from the sample by Trizol extraction following the manufacture’s protocol (Sigma-Aldrich) and the small RNAs were re-suspended in 7μL of DEPC-H_2_O. 6μL of the recovered, RPP-treated, purified small RNAs were used in the generation of small RNA libraries using the Truseq Small RNA sample preparation kit (Illumina). Samples were multiplexed and sequenced on an Illumina 2500.

**Small RNA analysis***. Trimming and removal of structural small RNAs*. Small RNA analysis was performed using Bowtie (1.1.1), custom Perl (5.16.3) scripts and Wormbase (WS240). Adapter sequences ('tggaattctcgggtgccaaggc') were removed from the 3' end of each read and filtered by size to obtain inserts 18-30 nt long, using Skewer ([7](#_ENREF_7)). Reads from each library that aligned with zero mismatches to the sense strand of structural small RNAs were removed ([4](#_ENREF_4)); sequences for 140 snoRNAs, 631 tRNAs, 1 scRNA, 22 rRNAs, 68 ncRNAs, and 114 snRNAs were extracted from WormBase and used in this analysis. The resultant nonstructural small RNA reads were used in the following analyses. The nonstructural small RNAs were aligned to *C. elegans* miRNAs obtained from WormBase (WS240) and used to normalize vsiRNA reads.

*Analysis of vsiRNA profiles*. Small RNA reads were aligned to the Orsay Virus genome (Genbank IDs: HM030970.2 and HM030971.2) using Bowtie and allowing zero mismatches essentially as described ([8](#_ENREF_8)). Aligned reads were then parsed by size, polarity, and 5' nucleotide identity using a custom perl script. For small RNA libraries cloned after RPP treatment, as many as 98% of the reads were antisense polarity. In order to not loose the information in the sense reads, the sense vsiRNAs were normalized to the total sense vsiRNAs ([9](#_ENREF_9)). Antisense reads were normalized to total vsiRNAs.

*Analysis of vsiRNA distribution.* Small RNA reads that aligned to the OrV genome were binned into groups of 20-nt, based on their mapping positions determined by either the 5’ ends of reads mapped to sense strand or the 3’ ends of reads mapped to antisense strand. Reads per million miRNAs (x1000) were graphed.

**Viral RNA detection by Northern blotting***.* Total RNA was extracted from homogenized worms using Trizol (Sigma-Aldrich) by following the manufacturer’s protocol, with the addition of an extra chloroform separation with a 1:1 ratio before precipitation. 4μg of high molecular weight RNA was ran on a 1.2% agarose gel with formaldehyde. P-32 labeled cDNA corresponding to OrV RNA1 was used to detect OrV infection, and a PCR fragment of gfp DNA was used to detect both RNA1 and RNA3 of FR1gfp. Hybridization of both probes was done at 65°C for three hours.

**FR1gfp induction and OrV infection***.* For detection of FR1gfp, animals were synchronized and grown at 20°C until they reached the L4 stage. L4 staged animals were heat shocked at 34°C for 4 hours and then returned to 20°C. eGFP was observed under a dissecting microscope 48 hours later. For OrV infections, 10-30 young adult worms were transferred to new 60mm NGM plates seeded with OP50 *E. coli*. 10 young adults are sufficient for the N2 strain; however more worms were used for difference mutants, due to a smaller brood size. 50μL of OrV filtrate was added to each plate and worms were incubated at 20°C. Five days post infection, the next generation of worms were collected for RNA extractions. OrV filtrate was prepared by growing infected *drh-1(tm1329)* animals. Adult worms were homogenized and centrifuged for 1 minute at 13,000rpm. The resulting supernatant was filtered with a 20μm filter and used in experiments.

**Identification of four novel alleles of *drh-1* by a mapping strategy based on whole genome sequencing.** We chose to map the causal mutations in *ucr2*, *ucr3*, *ucr4* and *ucr6* mutants. While these four mutants were proficient in exo-RNAi (Fig. 2), they produced clearly reduced levels of both the positive-strand primary vsiRNAs and the negative-strand secondary vsiRNAs compared to wild-type N2 worms, suggesting defective antiviral RNAi in these mutants. Compared to N2 worms, the positive-strand 23-nt primary vsiRNAs and the negative-strand 22-nt secondary vsiRNAs with 5’-terminal G (22G) were reduced respectively by 12.8% and 37.1% in *rrf-1* mutant and by 28.6% and 23.4% in *drh-1(tm1329)* (Table S1). Similarly, (+)23-nt and (-)22G vsiRNAs were reduced respectively by 7%-26% and 20.4%-30.1% in *ucr2*, *ucr3*, *ucr4* and *ucr6* mutants. Selection of the RNAi-proficient mutants for mapping would exclude these mutants identified in previous exogenous RNAi screens ([4](#_ENREF_4)). However, several attempts to map the *ucr* alleles by the classical map-based cloning approach ([10](#_ENREF_10)) were unsuccessful because the traditional mapping strain, CB4856, was unable to suppress replication of FR1gfp. This also prevented the use of an early mapping strategy by whole genome sequencing ([4](#_ENREF_4)), which requires the generation of a mapping interval. Therefore, we adopted a mapping-by-sequencing strategy developed recently in plants that identifies the causal mutations by whole-genome resequencing of pooled F2 progenies from a single backcross with the parental strain ([4](#_ENREF_4)).

Briefly, we backcrossed each mutant to the parental strain, N2;*FR1gfp,* and identified twenty F2 animals homozygous for the wildtype or mutant allele by scoring the segregation of the GFP phenotype in the F3 generation (Fig. S2). The F3 populations from each group were pooled for genome resequencing for at least 20x coverage (Table S2). The causal mutation was identified by interrogating the whole-genome resequencing data from each pair of wildtype and mutant pools using a custom computational pipeline for mutation calling by coverage depth and nucleotide frequency (mutCAN, available at https://github.com/JinfengLu/mutCAN.git)([11](#_ENREF_11)). We narrowed down the candidate SNPs unique to the mutant pool by firstly identifying only those mutations that could be EMS-induced (G→A or C→T) and alter amino acid sequence before filtering the remaining SNPs with a user defined allele frequency.

Unexpectedly, the final candidate SNPs for each of the four worm mutants included one in the *drh-1* gene on chromosome IV. Both *ucr3* and *ucr4* contained a missense mutation, leading to single amino acid substitution in the N- and C-terminal domains of DRH-1, respectively (Fig. S3A). The N-terminal domain of DRH-1 shares no detectable similarity to the N-terminal CARDs of mammalian RIG-I/MDA5. By contrast, the proline residue at position 966 (Pro^966^) mutated in *ucr4* is conserved in the C-terminal domain of both *C. elegans* DRH proteins and mammalian RIG-I/MDA5 (Fig. S3B). A nonsense mutation in *ucr6* induced a premature stop codon at residue position 865 of DRH-1, resulting in a putative protein that lacks the entire C-terminal domain (Fig. S3A).

The G→A transition identified in *ucr2* was located at the 5’-terminal nucleotide position of intron 13 immediately after exon 14 of *drh-1* and was predicted to disrupt the 5’ consensus splice site, 5’-AG|GURAGUU-3’ (Fig. S3A). Indeed, our characterization of the mature mRNA of *drh-1* in *ucr2* mutant worms revealed the use of an alternative, upstream splice site, 5’-GG|GUUUGUA, leading to a 90-nt deletion in the coding sequence (Fig. S3B/S3C). The resultant mRNA encodes a protein with a predicted in-frame deletion of 30 amino acids from position 596 to 625. This deletion would disrupt the Hel2i domain conserved in all of the known dra-ATPases (Fig. S3A) ([12](#_ENREF_12)).

The computational identification of the candidate SNPs in our approach used a relaxed allele frequency for the wildtype (0-40%) and mutant (60-100%) pools. In contrast, the mutant allele frequency was predicted as 0 for the wildtype pool and 100% for the mutant pool because the two phenotypically distinct pools of F3 families were selected for genome resequencing. To determine the actual frequency of the identified *drh-1* alleles, we individually genotyped the original F2s that made up the pools for *ucr4* and *ucr6*. The frequency of the specific *drh-1* mutant allele was 0 in the sequenced wild-type pool of both *ucr4* and *ucr6* mutants and the twenty F3 families in the wild-type pool of both mutants also were all genotyped as homozygotes for the wild-type allele. However, the frequency of the *ucr4* and *ucr6* alleles of *drh-1* obtained by sequencing the mutant pool of mutants was 83.7% and 75%, respectively, both of which were lower than the predicted value. Consistently, genotyping revealed that the twenty F2s in the mutant pool of either *ucr4* or *ucr6* included 17 homozygotes and 3 heterozygotes for the respective mutant allele, yielding a mutant allele frequency of 92.5% for both mutants. These results indicate that use of a relaxed allele frequency is necessary for the identification of candidate SNPs in genetic screens in which the mutant phenotype cannot be identified with 100% accuracy.

**Rescue of mutant alleles of *drh-1* by transgenic complementation.** A previous study ([13](#_ENREF_13)) has shown that a *DRH-1* transgene driven by the commonly used *sur-5* promoter, which is active in most of the worm cells (14), can rescue DRH-1 function in *drh-1* mutant worms. The same strategy was used to determine if the mutant phenotypes in these four mutants were indeed caused by the SNPs identified in *drh-1*. The *DRH-1* transgene was injected into the gonads of the mutant worms along with a *mCherry* marker transgene so that the two transgenes would form an extrachromosomal array and express red fluorescence in the pharynx of the progeny worms (Fig. S4A). Most of the resulting transgenic F1 worms randomly pass the transgene array onto only some of their next generations. Thus, we selected those worms free of the transgene array and the red fluorescence in the pharynx in the F2 population to serve as an internal negative control. For all of the four *drh-1* worm mutants, we detected the viral GFP expression only in animals that lacked red fluorescence from the transgene array (Fig. S4B). In contrast, all of the mutant animals that carried the *mCherry/DRH-1* transgene array, identified by red fluorescence in the pharynx tissue, did not exhibit green fluorescence from FR1gfp (Fig. S4B). Therefore, the transgenic expression of wildtype DRH-1 protein successfully restored antiviral RNAi, resulting in the rescued suppression of the viral GFP expression in all of the four worm mutants. In contrast, the mutant worms continued to support high levels of FR1gfp replication and GFP expression from the transcribed subgenomic RNA when they did not inherit the *DRH-1* transgene. The perfect correlation between the absence of the *DRH-1* transgene and the increased virus susceptibility in all of the four mutants demonstrates that the defective antiviral RNAi phenotype in *ucr2*, *ucr3*, *ucr4* and *ucr6* was caused by the SNPs identified in *drh-1*.

We further compared the viral replicon RNA levels by Northern blotting in *ucr2*, *ucr3*, *ucr4* and *ucr6* mutants with or without the *DRH-1* transgene. To this end, we isolated 10 red fluorescent animals that carried the transgene array from each mutant, induced FR1gfp replication when their progeny worms reached L4 stage and extracted total RNAs 48 hours after heat treatment. In all four mutants, both RNAs 1 and 3 of FR1gfp accumulated to markedly reduced levels following the ectopic expression of the *DRH-1* transgene (Fig. S4C), indicating suppression of FR1gfp replicon by the expression of the *DRH-1* transgene in *ucr2*, *ucr3*, *ucr4* and *ucr6* mutant worms. Extrachromosomal transgene arrays are passed onto next generations in different rates, which might explain why FR1gfp replicated to different levels in *ucr2*, *ucr3*, *ucr4* and *ucr6* progeny worms from their parents (Fig. S4C). Lower rates of the transgene array inheritance in *ucr3* and *ucr4* progeny worms would produce more worms to support FR1 replication due to the absence of the *DRH-1* transgene.

1. Lu R, Yigit E, Li WX, Ding SW. 2009. An RIG-I-Like RNA helicase mediates antiviral RNAi downstream of viral siRNA biogenesis in Caenorhabditis elegans. PLoS Pathog 5:e1000286.

2. Johnson KL, Ball LA. 1999. Induction and maintenance of autonomous flock house virus RNA1 replication. J Virol 73:7933-42.

3. Frokjaer-Jensen C, Davis MW, Hopkins CE, Newman BJ, Thummel JM, Olesen SP, Grunnet M, Jorgensen EM. 2008. Single-copy insertion of transgenes in Caenorhabditis elegans. Nat Genet 40:1375-83.

4. Gu W, Shirayama M, Conte D Jr, Vasale J, Batista PJ, Claycomb JM, Moresco JJ, Youngman EM, Keys J, Stoltz MJ, Chen CC, Chaves DA, Duan S, Kasschau KD, Fahlgren N, Yates JR 3rd, Mitani S, Carrington JC, Mello CC. 2009. Distinct argonaute-mediated 22G-RNA pathways direct genome surveillance in the C. elegans germline. Mol Cell 36(2):231-44

5. Jorgensen EM, Mango SE. 2002. The art and design of genetic screens: caenorhabditis elegans. Nat Rev Genet 3:356-69.

6. Pak J, Maniar JM, Mello CC, Fire A. 2012. Protection from feed-forward amplification in an amplified RNAi mechanism. Cell 151:885-99.

7. Jiang H, Lei R, Ding SW, Zhu S. 2014. Skewer: a fast and accurate adapter trimmer for next-generation sequencing paired-end reads. BMC Bioinformatics 15:182.

8. Li Y, Lu J, Han Y, Fan X, Ding SW. 2013. RNA interference functions as an antiviral immunity mechanism in mammals. Science 342:231-4.

9. Ashe A, Belicard T, Le Pen J, Sarkies P, Frezal L, Lehrbach NJ, Felix MA, Miska EA. 2013. A deletion polymorphism in the Caenorhabditis elegans RIG-I homolog disables viral RNA dicing and antiviral immunity. Elife 2:e00994.

10. Davis MW, Hammarlund M, Harrach T, Hullett P, Olsen S, Jorgensen EM. 2005. Rapid single nucleotide polymorphism mapping in C. elegans. BMC Genomics 6:118.

11. Guo Z, Lu JF, Wang XB, Zhan B, Li WX, Ding SW. 2017. Lipid flippases promote antiviral silencing and the biogenesis of viral and host siRNAs in Arabidopsis. Proc Natl Acad Sci U S A In press.

12. Luo D, Kohlway A, Pyle AM. 2013. Duplex RNA activated ATPases (DRAs): platforms for RNA sensing, signaling and processing. RNA Biol 10:111-20.

13. Guo X, Zhang R, Wang J, Ding SW, Lu R. 2013. Homologous RIG-I-like helicase proteins direct RNAi-mediated antiviral immunity in C. elegans by distinct mechanisms. Proc Natl Acad Sci U S A 110:16085-90.

14. Gu T, Orita S, Han M (1998) Caenorhabditis elegans SUR-5, a novel but conserved protein, negatively regulates LET-60 Ras activity during vulval induction. Mol Cell Biol

18(8):4556–4564.
